# Non-coding RNAs expression in SARS-CoV-2 infection: Pathogenesis, clinical significance and therapeutic targets

**DOI:** 10.1038/s41392-023-01669-0

**Published:** 2023-12-06

**Authors:** Xiaoxing Liu, Wandi Xiong, Maosen Ye, Tangsheng Lu, Kai Yuan, Suhua Chang, Ying Han, Yongxiang Wang, Lin Lu, Yanping Bao

**Affiliations:** 1 Peking University Sixth Hospital, Peking University Institute of Mental Health, NHC Key Laboratory of Mental Health (Peking University), National Clinical Research Center for Mental Disorders (Peking University Sixth Hospital), 100191 Beijing, China; 2 Peking-Tsinghua Center for Life Sciences and PKU-IDG/McGovern Institute for Brain Research, Peking University, 100871 Beijing, China; 3 Key Laboratory of Tropical Biological Resources of Ministry of Education, School of Pharmaceutical Sciences, Hainan University, 570228 Haikou, China; 4 Key Laboratory of Animal Models and Human Disease Mechanisms of the Chinese Academy of Sciences & Yunnan Province, KIZ/CUHK Joint Laboratory of Bioresources and Molecular Research in Common Diseases, Kunming Institute of Zoology, Chinese Academy of Sciences, 650204 Kunming, Yunnan China; 5 National Institute on Drug Dependence and Beijing Key Laboratory of Drug Dependence, Peking University, Beijing, 100191 China; 6 Institute of Brain Science and Brain-inspired Research, Shandong First Medical University & Shandong Academy of Medical Sciences, 250117 Jinan, Shandong China; 7 Department of Neurology, Shandong Provincial Hospital Affiliated to Shandong First Medical University, Jinan, Shandong China; 8 School of Public Health, Peking University, 100191 Beijing, China

**Keywords:** Epigenetics analysis, Predictive markers

## Abstract

The coronavirus disease 2019 (COVID-19) pandemic has been looming globally for three years, yet the diagnostic and treatment methods for COVID-19 are still undergoing extensive exploration, which holds paramount importance in mitigating future epidemics. Host non-coding RNAs (ncRNAs) display aberrations in the context of COVID-19. Specifically, microRNAs (miRNAs), long non-coding RNAs (lncRNAs), and circular RNAs (circRNAs) exhibit a close association with viral infection and disease progression. In this comprehensive review, an overview was presented of the expression profiles of host ncRNAs following SARS-CoV-2 invasion and of the potential functions in COVID-19 development, encompassing viral invasion, replication, immune response, and multiorgan deficits which include respiratory system, cardiac system, central nervous system, peripheral nervous system as well as long COVID. Furthermore, we provide an overview of several promising host ncRNA biomarkers for diverse clinical scenarios related to COVID-19, such as stratification biomarkers, prognostic biomarkers, and predictive biomarkers for treatment response. In addition, we also discuss the therapeutic potential of ncRNAs for COVID-19, presenting ncRNA-based strategies to facilitate the development of novel treatments. Through an in-depth analysis of the interplay between ncRNA and COVID-19 combined with our bioinformatic analysis, we hope to offer valuable insights into the stratification, prognosis, and treatment of COVID-19.

## Introduction

It is still a public health concern of the coronavirus disease 2019 (COVID-19) pandemic worldwide, three years after its outbreak. Until July 12, 2023, the global cumulative number of COVID-19 confirmed cases rose to 767.7 million and the cumulative death increased to nearly seven million, indicating that we must remain vigilant against COVID-19. Furthermore, the pandemic has inflicted a substantial attack to the global economy; most countries encountered negative gross domestic product rates in 2020 based on estimations by the World Bank and International Monetary Fund.^1^ The *World Economic Situation and Prospects*, an United Nations’ latest report, suggests that prospects for a robust global economic recovery remain bleak due to persistent repercussions of the COVID-19 pandemic.^2^ Therefore, it is still critical to focus attention on COVID-19.

Severe acute respiratory syndrome coronavirus 2 (SARS-CoV-2), a single-stranded positive ribonucleic acid (RNA) virus, is the causative agent, and multiple variants have emerged, ranging from alpha to omicron.^3^ SARS-CoV-2, belonging to the Sarbecovirus subgenus of betacoronavirus, possesses a genomic RNA with an average size of 26-32 kilobase and an outer shield composed of envelope (E), membrane (M), and spike (S) proteins.^4^ This virus primarily invades host cells through a combination with its receptor protein, angiotensin-converting enzyme 2 (ACE2), leading to immune system damage, and increased inflammatory factors release and even cytokine storms.^4,5^ The COVID-19 disease presents with a diverse kind of clinical symptoms affecting multiple systems, including respiratory, neuropsychiatric, cardiovascular, gastrointestinal, musculoskeletal and endocrine systems. It is common overserving fever, cough, shortness of breath and general malaise in these patients.^6,7^ In addition, a significant proportion of recovered individuals experience long-term symptoms referred to as “long COVID”, which is defined by the World Health Organization (WHO) as lasting for at least 2 months and cannot be explained by an alternative diagnosis occurring usually 3 months from the onset of COVID-19. These long-term symptoms may include fatigue, muscular weakness, dyspnea and neuropsychiatric manifestations such as depression, anxiety and cognitive deficits.^8,9^ Despite extensive global efforts dedicated to investigating SARS-CoV-2, our current understanding of its pathogenesis, including clinical progression and effective treatments, remains in progress.

**Fig. 1:**
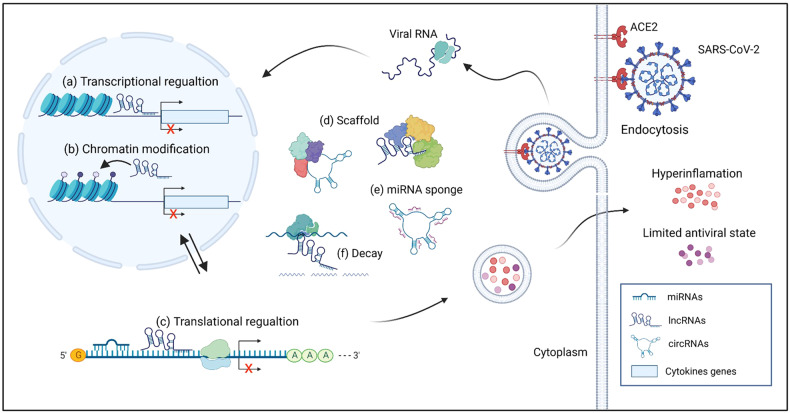
The main ways for ncRNAs in modulating gene expression in SARS-CoV-2-infected cells. The main mechanisms of the ways in which ncRNAs regulate gene expression are shown: **a** transcriptional regulation, **b** chromatin biology, **c** translational regulation, **d** scaffolding, **e** mRNA sponge, and **f** mRNA decay. Biorender was used to generate this figure

Non-coding RNAs (ncRNAs) serve as a critical regulator of the genome, providing an insight to viral pathogenesis and thus to developing antiviral therapeutics. NcRNAs constitute approximately 90% of RNAs in the human genome and participate in both physiological and pathological processes.^10^ Among these ncRNAs, the most studied types include microRNAs (miRNAs), long non-coding RNAs (lncRNAs), and circular RNAs (circRNAs).^11^ MiRNAs are a type of short ncRNAs that mediate genes and subsequent signaling by regulating the expression of other RNAs, especially messenger RNAs (mRNAs). In contrast, lncRNAs, a kind of ncRNAs with more than 200 nucleotides, can modulate the transcription of neighboring or distant genes, as well as regulate chromatin biology.^12^ The third major class of ncRNAs is circRNAs, a novel class with a closed continuous loop structure. Studies about the circRNAs function is still in progress, while many research have found that circRNAs can function as miRNA sponges, even binding multiple miRNA molecules and inhibit their roles.^13^ In a short brief, the ways in which these ncRNAs modulate gene expression can be summarized as follows (Fig. 1): (1) miRNA can target the mRNA to regulate the cascades; (2) some lncRNAs can make an impact on mRNA stability and manage the translation of related mRNAs in the cytoplasm; (3) some ncRNAs, such as lncRNAs and circRNAs, can function as scaffolds that enable interactions with multiple proteins; (4) some abundant circRNAs can bind miRNAs in the cytoplasm, acting as miRNA sponges to prevent miRNAs from binding their target mRNAs; and (5) some lncRNAs can recruit proteins to mRNAs and mediate mRNA decay.^14–16^ It is currently believed that ncRNAs may also play regulatory roles in the pathogenesis of COVID-19.

What is noteworthy is that both humans and viruses can produce ncRNAs, and virus-encoded ncRNAs can manipulate the host cell machinery to facilitate viral genome expression and protein production, as well as inhibit antiviral pathways.^17^ Extensive reviews have summarized the primary functions of virus-encoded miRNAs/lncRNAs/circRNAs, including regulating viral gene expression for latency control or activation transmission, and modulating the host immune system to create a favorable intracellular environment for viral persistence.^18–20^ Currently, virus-encoded ncRNAs are mainly identified in deoxyribonucleic acid (DNA) virus infections, while few originate from RNA viruses due to limited accessibility of host ncRNA biogenesis machinery in nuclei for RNA virus.^17^ However, some investigations have detected the virus-encoded ncRNAs expression after infection with various coronaviruses such as Middle East respiratory syndrome coronavirus (MERS-CoV), SARS-CoV-1 as well as SARS-CoV-2. For instance, Cai et al. discovered 3437 circRNAs derived from SARS-CoV-2 in 2020 which were associated with cholesterol metabolism processes and cellular responses to oxidative stress.^21^ Similarly, 40 viral miRNAs derived from the SARS-CoV-2 genome was identified, which mostly targeted genes of signaling pathway, epigenetics factors, tumor suppressors, transcription factors, and various kinases.^22^ Despite the significance of virus-encoded ncRNAs in modulating viral diseases and their potential clinical applications, the mechanisms underlying these ncRNAs, particularly those encoded by RNA viruses, remain uncertain. Therefore, we did not delve further into this topic in our review, which may represent a missing piece for comprehending the interplay between hosts and viruses.

Given the pivotal role of host ncRNAs in viral pathogenesis, there is promising potential to develop early identification, differentiation predictor, and efficacious interventions based on ncRNAs for managing the ongoing COVID-19 pandemic and preventing long-term sequelae. This review is specifically aimed at elucidating the expression profiles and functions of host ncRNAs (miRNAs, lncRNAs, and circRNAs) in SARS-CoV-2 pathogenesis, from viral invasion and replication to multiorgan deficits, and even to long COVID. Additionally, we provide a comprehensive overview of current research investigating the efficacy of ncRNA-based biomarkers and therapeutic approaches in relation to COVID-19 and long COVID.

## Dysregulated expression of the host ncRNAs in COVID-19

In the present, the main approaches for measuring ncRNAs expression can be divided into two types.^23^ One method with low throughput is represented by quantitative real-time polymerase chain reaction (RT-qPCR), northern blot, and in situ hybridization. Among these, RT-qPCR is due to its inexpensive and sensitive properties,^24^ now widely employed in clinical and basic research for detecting single or a small number of ncRNAs.^25^ Moreover, novel techniques have been developed based on the conventional PCR method, such as droplet-based digital PCR (ddPCR), which primarily relies on limited dilution, end-point PCR, and Poisson statistics.^26^ DdPCR offers several advantages including remarkable sensitivity and specificity, absolute quantification without a standard curve, excellent reproducibility, and high efficiency, making it a valuable addition to measuring the ncRNAs.^27^ The other type is a high-throughput technique such as RNA sequencing and microarrays. Compared with the microarrays which mainly pick up the known targets, the RNA sequencing can generate comprehensive and high-quality data that reveals unknown transcripts.^28^ Recently, the developing single-cell RNA sequencing and spatial transcriptomics sequencing technologies have made it possible that the RNAs expression in individual cells can be accurately distinguished within their native environment, exhibiting higher spatial specificity.^29^

Numerous studies have been conducted to measure the differentially expressed ncRNAs (DEncRNAs) in the host after SARS-CoV-2 infection through RT-qPCR, sequencing or microarrays (Table 1). Host miRNAs have garnered significant attention, with a plethora of published clinical reports investigating their presence in samples from individuals diagnosed with COVID-19. Despite the limited research on the expression profile of lncRNAs and circRNAs in the context of COVID-19, findings have revealed significant alterations in hundreds or even thousands of these ncRNAs among COVID-19 individuals. The majority of samples used to extract host ncRNAs were derived from various types of blood samples, including peripheral blood, peripheral venous blood, serum, plasma, red blood cell-depleted peripheral blood, and peripheral blood mononuclear cells (PBMCs).^17,30–34^ In addition, other tissues, such as nasopharyngeal samples, saliva, urine, bone, cerebrospinal fluid (CSF), and post-mortem lung biopsies, were also analyzed.^35–40^ Moreover, the expression profile of some ncRNAs can present dynamic changes over time, from the acute phase to post-acute, and even to the convalescence stage.^41,42^ In the following parts, we introduce the expression profiles of host miRNAs/lncRNAs/circRNAs after SARS-CoV-2 infection, and discuss the possible ways for SARS-CoV-2 to modulate the host ncRNAs expression.

**Table 1:** Clinical research on the detection of host ncRNAs expression in samples from people with SARS-CoV-2 infection

No.	Reference	Country	Participants	Sample size	Mean age	Sex (female, %)	Tissue type	Method	COVID-19 severity	Time for cases recruitment	Time of collecting samples	ncRNAs type
1	Abbasi-Kolli et al.^41^	Germany	COVID-19 cases	50	38.1	25, 50.00%	PBMCs	RT-PCR	/	2021.6-7	At acute infection, 6-7 weeks after the acute phase	miRNAs lncRNAs
			Health control	50	37.2	25, 50.00%						
2	Abdolahi et al.^217^	Iran	COVID-19 cases	30	59.67	10, 33.30%	PB	RT-qPCR	/	2020.5-8	At admission and discharge	miRNAs
			Health control	18	32.70	11, 61.10%						
3	Agwa et al.^32^	Egypt	COVID-19 cases	100	32.80	44, 44.00%	Serum	RT-qPCR	Mild, severe	2020	At admission	miRNAs
			Health control	100	34.30	48, 48.00%						lncRNAs
4	Akula et al.^187^	USA	COVID-19 cases	12	47.80	6, 50.00%	Plasma	NGS, RT-qPCR	Moderate-to-severe	/	/	miRNAs
			Health control	8	46.00	5, 62.50%						
5	Ayeldeen et al.^167^	Egypt	COVID-19 cases	200	58.96	98, 49.00%	Serum	RT-qPCR	Moderate, severe	/	/	miRNAs lncRNAs
			Health control	80								
6	Aznaourova et al.^57^	Germany	COVID-19 cases	11	70.36	3, 27.27%	PBMCs	scRNA-seq	Severe	/	/	lncRNAs
			Health control	8	57.50	4, 50.00%						
7	Bagheri-Hosseinabadi et al.^30^	Iran	COVID-19 cases	33	62.40	20, 60.60%	PB	RT-qPCR	/	/	At admission	miRNAs
			Health control	29	56.60	20, 69.00%						
8	Centa et al.^40^	Brazil	COVID-19 non-survivors	9	73.40	3, 33.30%	FFPE *post-mortem* lung biopsies	RT-qPCR	/	/	/	miRNAs
			Patients who died due to other causes	10	42.30	3, 30.00%						
9	Cheng et al.^54^	China	COVID-19 cases	29	Severe:74.00; NS: 69.14	/	PBMCs	NGS	Mild/moderate, severe	2020.3-4	/	miRNAs lncRNAs
			Health control	10	/	/						
10	de Gonzalo-Calvo et al.^88^	Spain	COVID-19 cases	79	68.00	35, 44.30%	Plasma	RT-qPCR	/	2020.3-5	At admission	miRNAs
11	Demiray et al.^218^	Turkey	COVID-19 cases	40	55.00	17, 42.50%	Serum	RT-qPCR	Mild, severe	2020.3-4	/	miRNAs
			Health control	10	36.00	6, 60.00%						
12	Devados et al.^113^	USA	COVID-19 cases	20	/	7, 35.00%	Nasopharyngeal swabs	/	Symptomatic	/	/	lncRNAs
13	Donyavi et al.^45^	Iran	COVID-19 cases	18	38.20	9, 50.00%	PBMCs	RT-qPCR	/	2020.4-7	At the acute period and the recovery period	miRNAs
			Health control	15	36.60	7, 47.70%						
14	Duecker et al.^44^	Germany	COVID-19 cases	21	72.00	4, 19.00%	Whole blood	NGS	Moderate, severe	/	At the acute period (day 0–3) and in the later course of the disease (>7 days)	miRNAs
			Health control	8	68.00	3, 37.50%						
15	Farr et al.^52^	Australia	COVID-19 cases	10	53.50	6, 60.00%	Plasma	NGS; RT-qPCR	Moderate. Severe	2020.2-4	After 2–15 days (average 8 days) of disease onset	miRNAs
			Health control	10	53.00	6, 60.00%						
16	Fayyad-Kazan et al.^219^	Lebanon	COVID-19 cases	12	/	/	Plasma	MiRCURY LNA miRNA miRNome qPCR	Mild, moderate, severe	/	At the time of diagnosis	miRNAs
			Health control	12	/	/						
17	Fernández-Pato et al.^43^	Spain	COVID-19 cases	96	Mild: 63.40; moderate: 59.40; severe: 66.20	43, 44.80%	Plasma	NGS	Asymptomatic/mild, moderate, severe	2020.3-8	At hospital entry or within the first days after hospitalization	miRNAs
			Health control	13	66.70	6, 46.20%						
18	Firoozi et al.^114^	Iran	COVID-19 cases	100	/	43, 43.00%	PBMCs	RT-qPCR	Asymptomatic, symptomatic	/	/	miRNAs circRNAs
			Health control	50	43.16	23, 46.00%						
19	Gambardella et al.^220^	USA	COVID-19 with or without CBV	321	61.90	145, 45.20%	EC-EVs	/	/	2020.10-2021.4	/	miRNAs
			Patients with CBV	37	65.37	15, 40.50%						
			Healthy control	57	59.4	28, 49.1%						
20	Garg et al.^46^	Germany	COVID-19 cases	38	DC: 59.00; VC: 59.50	7, 18.40%	Serum	RT-qPCR	Severe	/	/	miRNAs
			Influenza-ARDS patients	13	56.00	2, 15.40%						
			Health control	15	31.00	1, 6.70%						
21	Giuliani et al.^47^	Italy	COVID-19 cases	128	DC: 75.40; VC: 86.50	83, 64.80%	Serum	NGS	/	2020.3-6	At admission	miRNAs
22	Grehl et al.^221^	Germany	COVID-19 cases	10	/	6, 60.00%	Plasma	NGS	Mild, severe	2020.3-8	Within the first 3 weeks after symptom onset	miRNAs
			Patients with bacterial pneumonia	1	/	1, 100%						
			Health control	1	/	1, 100%						
23	Gutmann et al.^49^	UK	COVID-19 cases	97	/	36, 37.10%	Plasma	RT-qPCR; NGS	Mild, moderate, severe	2020.5-12	Mild/moderate: at admission; severe: within ICU admission	miRNAs
			Health control	11	40.00	6, 54.50%						
24	Haroun et al.^160^	Egypt	COVID-19 cases	150	49.43	61, 40.70%	Plasma	RT-qPCR	Moderate, severe	/	At admission	miRNAs
			Health control	50	45.80	/						
25	Keikha et al.^176^	Iran	COVID-19 cases	103	/	53, 51.50%	Serum	RT-qPCR	/	2020.12-2021.3	At admission	miRNAs
			Health control	20	/	/						
26	Li CX et al.^222^	China	COVID-19 cases	10	44.90	6, 60.00%	PB	NGS	Mild or moderate	2020.2-3	Within 1 week after diagnosis	miRNAs
			Health control	4	44.75	2, 50.00%						
27	Li CX et al.^53^	China	COVID-19 cases	10	44.90	6, 60.00%	PB	NGS	/	2020.2-3	/	miRNAs
			Health control	4	34.75	3, 75.00%						lncRNAs
28	Liu et al.^33^	China	COVID-19 cases	10	/	5, 50.00%	Red blood cell-depleted peripheral blood	RT-qPCR	Moderate, severe	2020.1–3	/	miRNAs
			Health control	4	/	/						
29	Loretelli et al.^146^	Italy	COVID-19 cases	57	54.70	29, 50.88%	PBMCs	qPCR	/	2020.3-10	/	miRNAs
			Post-COVID-19 cases	39	55.30	10, 25,64%						
			Health control	43	47.30	25, 58.14%						
30	Martı´nez-Fleta et al.^223^	Spain	COVID-19 cases	123	DC: 59.50; VC: 64.00	65, 52.80%	Plasma	RT-qPCR	Mild, severe	2020.3-4	Within 5 days upon admission	miRNAs
			CAP adult patients	33	Dc: 62.00; vc: 66.50	15, 45.50%						
31	McDonald et al.^37^	USA	COVID-19 cases	50	/	/	Serum, nasopharyngeal, urine	/	/	2020.3-5	/	miRNAs
			Negative control	25	/	/						
			Common cold coronavirus	6	/	/						
			Coronavirus NL63	6	/	/						
			Healthy control	11	/	/						
32	Meidert et al.^224^	Germany	COVID-19 cases	30	COVID-19 pneumonia: 63.00; COVID-19 ARDS: 65.00	4, 13.30%	EVs	NGS, RT-qPCR	/	2020.3-4	At admission	miRNAs
			Health control	18	35.00	7, 38.90%						
			CAP	12	71.00	4, 33.30%						
33	Mi et al.^38^	China	COVID-19 cases with fracture	30	/	/	Muscle, bone, and bone marrow specimens	RT-qPCR	/	/	At the time of diagnosis	miRNAs
			Fracture patients	50	55.90	25, 50%						
34	Parray et al.^31^	Sweden	COVID-19 cases	29	Asymptomatic: 54.50; mild: 49.60; severe: 58.22	0, 0%	PVB	Affymetrix GeneChip miRNA 4.0 array	Severe, mild, and asymptomatic	/	At the time of diagnosis prior to isolation, or at admission	miRNAs
35	Pimenta et al.^181^	Brazil	COVID-19 cases	72	/	/	Saliva	RT-qPCR	symptomatic clinical conditions with no indication of hospitalization, symptomatic clinical conditions with respiratory disorders, severe	2020.6-10	At the time of diagnosis	miRNAs
			Health control	39	/	/						
36	Reinhold et al.^39^	Germany	COVID-19 cases	38	68.60	10, 26.32%	Serum, CSF	NGS	/	/	/	lncRNAs circRNAs
			HSVE patients	10	54.60	4, 40.00%						
			Patients with non-inflammatory, non-neurodegenerative neurological diseases	28	58.70	11, 39.29%						
37	Rodrigues et al.^36^	Brazil	COVID-19 cases	18	41.50	/	Nasopharyngeal swab, saliva	RT-qPCR	/	/	/	lncRNAs
			Healthy control	23	40.00	/						
38	Rombauts et al.^55^	Spain	COVID-19 cases	60	63.00	23, 38.30%	PB	NGS	with ARDS, without ARDS	202.3-7	At admission and on day 7 of hospital admission	lncRNAs
39	Srivastava et al.^225^	India	COVID-19 cases	17	/	/	PB	NGS	Moderate, severe	/	/	miRNAs
			COVID-19 non-survivors	16	/	/						
			Healthy control	10	/	/						
40	Taheri et al.^161^	Tehran	COVID-19 cases	91	57.18	38, 41.8%	PB	RT-qPCR	ICU, non-ICU	2020.3-4	At admission	lncRNAs
			Healthy control	91	/	39, 42.9%						
41	Tang et al.^50^	China	COVID-19 cases	12	/	9, 75.00%	Red blood cell-depleted whole blood	NGS	Moderate, severe	/	At admission	miRNAs
			Healthy control	4	/	2, 50.00%						lncRNAs
42	Wang et al.^226^	China	COVID-19 cases	37	53.90	11, 29.70%	Whole blood	NGS	Mild, severe	2020.1-2	At admission	miRNAs
			Healthy control	8	45.80	1, 12.50%						lncRNAs
43	Wilson et al.^48^	UK	COVID-19 cases	58	63.00	22, 37.90%	Plasma	NGS	Mild, moderate, severe	2020.4-10	At admission	miRNAs
			Healthy control	/	/	/						
44	Wu J et al.^51^	China	COVID-19 cases	29	47.45	12, 41.40%	Plasma	RT-qPCR	Mild, severe	2020.1-5	At admission	miRNAs
			Healthy control	29	48.34	15, 51.70%						
45	Wu WZ et al.^35^	China	COVID-19 cases	4	54.30	/	Nasopharyngeal swabs	NGS	/	2020.4	At admission	miRNAs
			Healthy control	4	50.50	/						
46	Wu YP et al.^34^	China	Recurrent COVID-19 cases	3	/	/	Whole blood	/	/	/	At admission	lncRNAs circRNAs
			Healthy control	3	/	/						
47	Yang et al.^227^	China	COVID-19 cases	5	/	/	Whole blood	/	/	2020.1-2	At admission	lncRNAs
			Healthy control	3	/	/						
48	Zhang et al.^228^	China	COVID-19 cases	39	/	17, 43.59%	PBMCs	Arraystar Human LncRNA Microarray V5.0	Mild, severe	2020.1-2	/	lncRNAs
			Healthy control	5	/	/						
49	Zheng et al.^42^	China	COVID-19 cases	18	/	7, 38.90%	PVB	NGS	Mild, moderate, severe	2020.1-4	At the time of treatment, convalescence stage, rehabilitation stage	miRNAs lncRNAs

*ARDS* associated acute respiratory distress syndrome, *CAP* community-acquired pneumonia, *CBV* cerebrovascular, *CSF* cerebrospinal fluid, *DC* discovery cohort, *EC-EVs* endothelial cells-extracellular vesicles, *EVs* extracellular vesicles, *FC* fold change, *FFPE* formalin-fixed paraffin-embedded, *HSVE* herpes simplex virus type 1 encephalitis, *NGS* next-generation sequencing, *NS* non-severe, *PB* peripheral blood, *PBMCs* peripheral blood mononuclear cells, *PVB* peripheral venous blood, *RT-qPCR* quantitative real-time polymerase chain reaction, *scRNA-seq* single-cell RNA sequencing, *VC* validation cohort

### SARS-CoV-2-associated DEncRNAs

#### miRNAs

A number of investigations have reported that the host miRNAs expression is altered in COVID-19 individuals in contrast to healthy controls, suggesting potential involvement of miRNAs in the COVID-19 pathogenesis.^43^ In addition, the host miRNAs expression can be influenced by disease severity, as evidenced by comparisons between asymptomatic/mild and symptomatic patients or among mild, moderate, and severe cases, highlighting the remarkable potential of miRNAs in distinguishing the COVID-19 severity through demonstrating the diverse landscape of miRNAs in patients with varying disease severities.^31^ In addition, the temporal sensitivity of the differentially expressed miRNAs (DEmiRNAs) profile in the context of COVID-19 is evident. On one hand, it exhibits dynamic changes within a few days of disease onset, even changing between the acute stage (within 3 days) and later period (>7 days), thereby indicating the potential to predict symptoms characterized by rapid onset after SARS-CoV-2 infection.^44,45^ On a broader temporal scale encompassing treatment, convalescence, and rehabilitation stages, the expression pattern of DEmiRNAs which resulted from SARS-CoV-2 infection also demonstrates discernibility.^42^ Furthermore, SARS-CoV-2 infection may lead to a distinct expression profile of host miRNAs differentiated from those infected with other viruses. Compared to patients with influenza-associated acute respiratory distress syndrome (influenza-ARDS), three upregulated miRNAs in the serum were identified in severe COVID-19 patients, contributing to deciphering the unique pathogenesis of SARS-CoV-2.^46^

Among the myriad of dysregulated miRNAs, certain ones have undergone additional validation in diverse populations or through alternative methodologies.^46–48^ Further, the abundance of PCR or sequencing data facilitated a comprehensive analysis of the expression profile of DEmiRNAs in COVID-19 patients. This enabled us to identify DEmiRNAs that were repeatedly measured across multiple studies and compare their expression patterns among healthy controls, non-severe COVID-19 patients, and severe cases (Supplementary Tables S1–3). Notably, certain miRNAs such as miR-1246 and miR-106b-5p exhibited consistent differential expression across various studies, providing further validation for the impact exerted by SARS-CoV-2 on host miRNA landscape.^43,49–52^

#### lncRNAs

Differentially expressed lncRNAs (DElncRNAs) have been recognized in COVID-19 patients with varying disease severities in comparison with healthy controls, as well as among COVID-19 cases with different disease severities, indicating the potential involvement of lncRNAs in the pathogenesis of this disease.^53,54^ The expression of host lncRNAs also exhibited temporal sensitivity, either within an acute time frame (e.g., from admission to 7 days later) or over a longer progression period (e.g., during treatment, convalescence, and rehabilitation).^42,55^ Moreover, DElncRNAs can be detected in the recurrent COVID-19 cases, evidenced by that nearly one thousand DElncRNAs were identified in the recurrent COVID-19 patients compared with the healthy controls.^34^ However, further investigations are worthy to determine whether there exist differences in lncRNAs expression between individuals with a single SARS-CoV-2 infection and those experiencing reinfection, as the number of infections may increase the risk and disease burden, suggesting potential underlying distinctions.^56^

Insufficient research has been conducted to validate the expression profile of lncRNAs in COVID-19 cases, despite the identification of DElncRNAs at a cellular level. There was a study using single-cell RNA sequencing showing the DElncRNAs in the blood leukocytes in severe COVID-19 cases in comparison with the healthy control, revealing the possible involvement of lncRNAs in the disease development at a much finer spatial scale.^57^

#### circRNAs

The current research on differentially expressed circRNAs (DEcircRNAs) in COVID-19 is limited, yet it may offer valuable insights into the DEcircRNAs following SARS-CoV-2 infection and their presence across various samples from COVID-19 patients. In the human lung epithelial cells infected with SARS-CoV-2, more than five thousand circRNAs at various genomic location were identified via genome-wide dynamic analysis.^58^ In addition, in the whole-blood sample from recurrent COVID-19 cases, DEcircRNAs were also identified compared to the healthy control.^34^ The aforementioned investigations suggest that the SARS-CoV-2 infection can disrupt the expression of host circRNAs in blood, while a consistent dysregulation has also been observed in the CSF, evidenced by a differential expression profile of circRNAs identified in the CSF compared among COVID-19 case, healthy controls, and cases with neurological disease.^39^ Considering the tissue- and cell-specificity of the circRNAs expression, whether there is an overlap or difference in the types of DEcircRNAs between the neural cells and blood cells also needs more investigations for deeper understanding the systemic effects of SARS-CoV-2, such as using the single-cell RNA sequencing and spatial transcriptomics sequencing.

### Ways for SARS-CoV-2 to alter host ncRNAs expression

Numerous reviews have provided detailed explanations on the biogenesis of host miRNAs/lncRNAs/circRNAs.^10,12,59,60^ Most host miRNAs are generated through the canonical pathway, which involves transcription by RNA polymerase II or III and maturation via endonucleolytic processing. In addition, some miRNAs can be produced through noncanonical pathways where they are transcribed from short hairpin introns.^11^ The biogenesis of lncRNAs occurs in the nucleus and originates from the lncRNA genes. Depending on the canonical pathway, lncRNA species are transcribed by polymerase II or III and mature into transcripts.^12^ While the mechanism of circRNA biogenesis remains unclear, some studies have shown that it also relies on the canonical splicing machinery and most circRNAs are transcribed from known protein-coding regions.^60^

Following SARS-CoV-2 infection, there are differential expressions of massive host miRNAs, lncRNAs, and circRNAs, indicating that the virus invasion influences ncRNAs expression. However, the mechanisms by which the virus affects host ncRNA expression remain poorly understood due to challenges in defining boundaries between viral factors and host antiviral responses that can result in changes to ncRNA expression.^61^ Nevertheless, some reports suggest that viruses can directly target specific subsets of ncRNAs or impact global levels of ncRNA expression by influencing various aspects of host ncRNA biogenesis such as transcription, Dicer processing, and export.^62^ In a study involving SARS-CoV infected cells, a competing endogenous RNAs (ceRNAs) network was identified.^63^ Among these, the involvement of one mRNA antiviral innate immune response receptor RIG-1 (Ddx58) in the processes of mRNA splicing and miRNA biogenesis results in reprogramming of miRNA splicing and decreased miRNA expression when it is upregulated. Although this study suggests a potential mechanism for SARS-CoV-2 disruption of host ncRNAs through regulating certain key components involved in the miRNA splicing, it is likely that the actual mechanisms are more intricate. In 2023, Garnier et al. found no significant changes in nasopharyngeal swab specimens between COVID-19 patients and controls nor between severe and non-severe cases regarding the miRNA expression levels of several key proteins involved in miRNA biogenesis including protein argonaute-2 (*AGO2*), endoribonuclease dicer (*DICER1*), DiGeorge syndrome critical region 8 (*DGCR8*), drosha ribonuclease III (*DROSHA*), and *Exportin-5.*^64^ Consistently, in vitro experiments using normal human bronchial epithelial (NHBE) and Calu-3 cells invaded by SARS-CoV-2 also exhibited no alterations in these mRNA expressions. These findings suggest that SARS-CoV-2 infection had no effect on the mRNAs expression of key genes associated with miRNA biogenesis. Further investigations are warranted to elucidate the mechanisms by which SARS-CoV-2 induces alterations in host DEncRNAs expression.

## The roles of ncRNAs in COVID-19

The COVID-19 pathogenesis initiates with the invasion of SARS-CoV-2. In the early stages of infection, the viral S protein specifically binds to ACE2 receptors present in nasal and bronchial epithelial cells as well as pneumocytes. Mostly utilizing type 2 transmembrane serine protease (TMPRSS2) within these targeted cells, the virus gains entry through endocytosis into host cells.^65^ Subsequently, it manipulates the host cell machinery to replicate its RNA and assemble additional virions, leading to an escalation in viral copy numbers within the lower respiratory tract.^6^ Concurrently, infected cells and alveolar macrophages release inflammatory molecules while lymphocytes, monocytes, and neutrophils are recruited. In addition, lymphopoiesis impairment also occurs alongside increased cell apoptosis. During later stage, accelerated viral replication allows SARS-CoV-2 to invade pulmonary capillary endothelial cells, which intensifies inflammation and disrupts endothelial barriers. Furthermore, pulmonary edema can fill alveolar spaces, resembling early-phase ARDS symptoms.^66^

The development of viral sepsis, which may subsequently lead to multiorgan dysfunction, is a matter of greater concern. Severe lung injury, such as ARDS, represents the primary complication induced by SARS-CoV-2. It has been observed that 15–30% of hospitalized COVID-19 individuals will progress to develop COVID-19-associated ARDS.^67^ In addition, a range of complications have been demonstrated, including thrombotic events, myocardial dysfunction, and arrhythmia, as well as neuropsychiatric disorders.^68^ Despite the unclear mechanisms underlying these complications in the context of COVID-19, current perspectives on the pathophysiology of multiorgan failure following SARS-CoV-2 infection primarily focus on direct viral tissue damage and dysregulated host responses induced by the virus.^68^

**Fig. 2:**
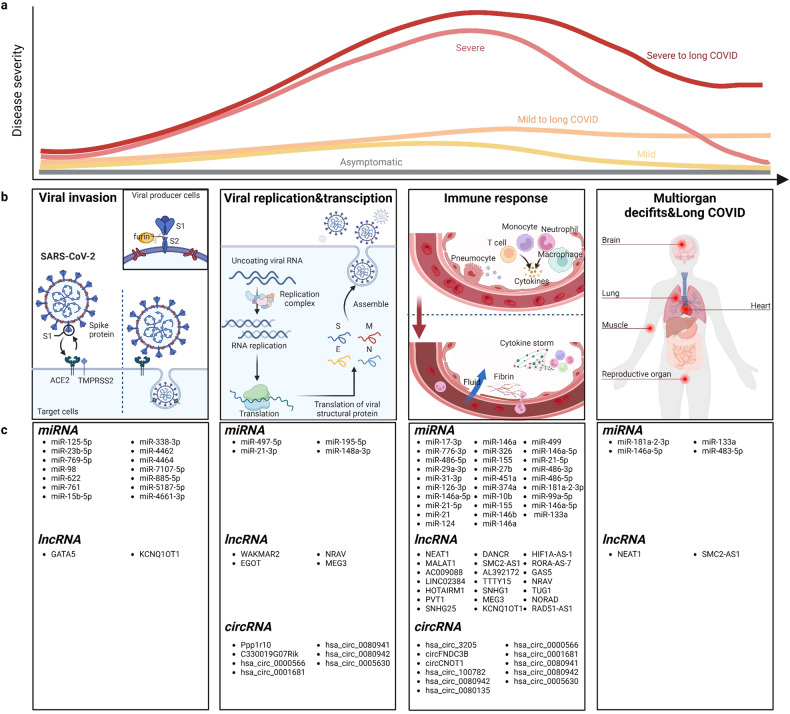
The functions of host ncRNAs in the pathogenesis of COVID-19 and representative ncRNAs. **a** Possible COVID-19 disease course is depicted as differentially colored curves of disease severity over time.^216^ **b** During the progression of this disease, viral invasion serves as the initial step. Following cleavage of the S protein into S1 and S2 subunits by furin in viral producer cells, the SARS-CoV-2 virus can effectively bind to the ACE2 receptor. Subsequent cleavage occurs either through TMPRSS2 or via endocytosis into the endolysosome. Upon entry into the cytoplasm, it undergoes replication to generate multiple copies for dissemination within the host organism. This is accompanied by translation of viral proteins or polypeptides, assembly, and eventual release into extracellular spaces. The released virus can undergo further reorganization, triggering release of inflammatory signaling molecules from infected cells and alveolar macrophages, while also recruiting T cells, monocytes, and neutrophils. Disease exacerbation leads to increased fluid accumulation in alveolar spaces and potentially cytokine storms that induce hyperinflammation. In late stages of illness, some patients may exhibit acute or persistent multiorgan deficits involving organs such as the brain, lungs, and heart. **c** In the context of COVID-19, several host miRNAs/lncRNAs/circRNAs have been identified through wet-lab experiments or bioinformatic analysis, elucidating their roles in various aspects of COVID-19 pathogenesis, encompassing viral invasion, replication, immune response modulation, multiorgan failure and long COVID. Biorender was used to generate this figure

Many studies have demonstrated that host ncRNAs exhibit differential expression patterns following SARS-CoV-2 infection but also play a crucial role in various aspects of its pathogenesis. Investigating the functions of ncRNAs in this process can significantly facilitate our understanding of the interplay between the SARS-CoV-2 and host. In the subsequent section, we present some prominent examples of host ncRNAs which have been identified with wet-lab or bioinformatic analysis in the context of COVID-19 (Supplementary Table S4), providing a discussion about how host ncRNAs contribute to COVID-19 pathogenesis through an intricate interplay via direct or indirect action and anti- or pro-viral effects, including viral invasion, replication, immune response modulation, multiorgan deficits as well as long COVID (Fig. 2).

### Impact on SARS-CoV-2 invasion

The invasion of SARS-CoV-2 into target cells involves more than just the interaction between the S protein and its receptor ACE2. The crucial step for virus is to enter the cellular membrane and reach the cytoplasm of targeted cells. To overcome the inherent repulsion between the virus and cell membrane, the S protein must transition into a metastable state (a state prone to transformation to a lower-energy state) before membrane fusion.^4^ Unlike SARS-CoV which relies on protease cleavage in target cells for this transition, in SARS-CoV-2 cleavage of the S protein occurs in two steps - first by furin in virus-producer cells (cleaving the S protein into S1 and S2 subunit) and then by proteases in target cells (cleaving at the S2 subunit). During the second cleavage, there are two routines utilized by SARS-CoV-2: (1) one dependent on TMPRSS2 located on plasma membranes of target cells and (2) the other relying on cathepsin L located within endolysosome of target cells.^4^ In host cells co-expressing ACE2 and TMPRSS2, TMPRSS2 is responsible for cleavage of the S2 subunit, followed by ACE2-mediated endocytosis that facilitates viral RNA release into the cytoplasm for replication and uncoating.^69,70^ However, in cases of inadequate TMPRSS2 expression or absence of virus-ACE2 complex interaction with TMPRSS2, SARS-CoV-2 can be internalized through endocytosis into the endolysosome where cathepsin L cleaves at the S2 subunit, leading to membrane fusion and subsequent viral RNA release.^71,72^

Despite the evidence from wet-lab about the role of host ncRNAs in regulating the SARS-CoV-2 invasion are still scant, some bioinformatics results indicate that some miRNAs and lncRNAs may modulate the entry process via targeting the furin, ACE2 and TMPRSS2.

#### Furin

Furin is a type I transmembrane protein that serves as a proprotein convertase, ubiquitously expressed in pulmonary, hepatic, and intestinal tissues.^73^ In the context of COVID-19, furin-mediated cleavage of the S1/S2 boundary is essential for virus-induced membrane fusion. This unique feature differentiates SARS-CoV-2 from other Sarbecovirus and enables zoonotic transfer to humans.^74^ A mutant SARS-CoV-2, which was lack of the furin cleavage site, exhibited reduced S protein processing in infected cells compared to parental SARS-CoV-2.^75^ Although direct experimental evidence on the role of host ncRNAs in modulating furin after SARS-CoV-2 infection is lacking, a bioinformatics study predicted that some host DEmiRNAs from infected cells can target FURIN mRNA, which may be regulated by SARS-CoV-2 to create a favorable environment for viral invasion.^76^

#### ACE2

ACE2 is the main receptor for SARS-CoV-2 entry, while its primary function in normal physiology is to convert angiotensin I and angiotensin II into angiotensin-(1-9) and angiotensin-(1-7), respectively.^77^ In the lower lung, type II alveolar cells is the major location where the ACE2 expresses, while the ACE2 expression is more pronounced in the upper bronchial epithelia and significantly elevated in the nasal epithelium, particularly within the ciliated cells.^78^ This distribution pattern aligns with the infection gradient of SARS-CoV-2, wherein nasal ciliated cells primarily serve as primary targets for viral infection during early stage.^79^ Severe COVID-19 patients possibly exhibit increased expression of ACE2 due to certain inflammatory cytokines such as interleukin-1β (IL-1β) and type I and II interferons (IFNs), and the result is the establishment of a positive-feedback loop, which facilitates viral replication.^79,80^

There are some ncRNAs serving antiviral role through inhibiting the ACE2. MiR-1246, which shares homology with ACE2 and targets its coding DNA sequence, has been identified as a negative modulator of ACE2 expression.^81^ Several studies have reported consistent upregulation of miR-1246 in the plasma of COVID-19 patients in comparison with healthy controls.^43,51^ Furthermore, miR-1246 expression level may increase with COVID-19 severity, as evidenced by consistent upregulation in severe patients compared to non-severe patients,^31,43,50^ indicating that with COVID-19 exacerbation, miR-1246 expression may gradually increase to prevent ACE2 expression and inhibit the viral invasion.^82^ In addition, the increased miR-200c-3p may directly target the 3’ untranslated region (UTR) of ACE2 and inhibit its expression in the epithelial cells.^50,52,82^ Similarly, other downregulated miRNAs namely miR-125-5P, miR-23b-5p and miR-769-5p can binds with 3’ UTR of ACE2 to block the virus entry and attachment.^83^

In line with the miRNAs changes, there were a lot of dysregulated DElncRNAs involved in SARS-CoV-2 invasion. The lncRNA GATA-binding protein 5 (*GATA5)*, which is significantly elevated in severe cases, can also inhibit ACE2 gene expression to block the virus entry into host cells.^54^

#### TMPRSS2

The TMPRSS2 protein is classified as a type II transmembrane protein and exhibits serine protease activity. However, its precise physiological function remain poorly understood. Inhibition of TMPRSS2 through a small-molecule protease inhibitor can significantly prevent SARS-CoV-2 entry in both human lung epithelial cells and a transgenic mouse model of severe COVID-19, demonstrating the essential role of TMPRSS2 in SARS-CoV-2 invasion.^84^ In comparison with healthy controls, there was a marked increase in the expression of TMPRSS2 in lung epithelial cells from COVID-19 patients, particularly club and ciliated cells.^85^ Despite that the evidence about the relationship between host ncRNAs and TMPRSS2 after SASR-CoV-2 infection is still limited, it has been mechanistically validated that miR-98 can directly bind the 3’ UTR of TMPRSS2 in human endothelial cells including human lung microvascular endothelial cells and human umbilical vein endothelial cells, and subsequently can block the virus entry.^86^

### Impact on SARS-CoV-2 replication

The overall life circle of SARS-CoV-2 includes not only the viral entry and endocytosis, but also the viral replication. After entry into the host cells, the virus produces multiple copies to spread inside the host body, following by the translation of the viral proteins or polypeptides. The SARS-CoV-2 genome harbors multiple open-reading frames (ORFs), encoding 16 nonstructural proteins (nsp 1–16) and necessitating the involvement of numerous proteins to sustain its replication cycle.^87^ Similar as the mRNAs, the viral genome also includes a 5’ cap structure along with a 3’ poly (A). Of these, the 5’ cap structure contains the sequence and UTR with stem-loop structures for RNA replication and translation. The 3’ UTR also embraces the structures required for viral RNA replication. In addition, the genome structure, 5’-UTR-replicase-S-E-M-N-3’ UTR-poly (A) tail, can create a suitable environment for virus replication and transcription.

The host ncRNAs have been highlighted in modulating the replication of SARS-CoV-2, possibly through directly targeting the viral genome or through virus-mediated alteration in the host transcriptome. Plasma miRNA profiling showed that the host miR-148a-3p can target virus genome, binding to the ORF1a, E, S and M genes and affecting virus entry and replication.^88^ Several downregulated miRNAs, including miR-497-5p, miR-21-3p and miR-195-5p, can target the coding strand of SARS-CoV-2, and lately inhibit its replication.^89,90^ In addition, some regulatory networks are controlled by the circRNA/lncRNA-miRNA-mRNA regulatory axis in the infected cells. Relevant research has reorganized a quintuple regulatory network including one miRNA (miR-124-3p), two circRNAs (ppp1r10 and C330019G07Rik) one lncRNA (Gm26917) and one hub gene Ddx58 in SARS-CoV cells. Ppp1r10 and C330019G07Rik can act as sponges for miR-124-3p to suppress Ddx58 degradation, resulting in the reduction of SARS-CoV-2 replication.^63^

### Impact on immune response to SARS-CoV-2

Uncontrolled viral replication may trigger multiple immunopathologic conditions in host cells. As a result, SARS-CoV-2 can effectively inhibit or delay the induction or function of type I and III IFNs by infected cells, thereby circumventing or postponing the onset of intracellular innate immune responses and contributing to immunopathology.^91–93^ This temporal delay in innate immune response is sufficient to cause asymptomatic infection or clinically mild disease, as T cells and antibody responses can develop and control the infection.^94,95^ However, if there is a prolonged delay in priming the adaptive immune response due to impaired innate immunity, SARS-CoV-2 could undergo extensive replication in the upper respiratory tract and lungs. Consequently, innate immunity takes over adaptive immunity by amplifying its response to control the virus but leads to elevated levels of innate cytokine/chemokine molecules, triggering a phenomenon known as “cytokine storm”, along with dysregulated innate and adaptive immune cells observed in severe and critical disease.^95,96^ During this process, certain inflammasomes such as Nod-like receptor family pyrin domain-containing 3 (NLRP3) inflammasome would be activated, inducing release of pro-inflammatory factors and cell death.^97^

Host ncRNAs play a crucial function in regulating the innate and adaptive immune response.^98–100^ Although miRNA, lncRNA, and circRNA have distinct mechanisms of action, their roles in the host immune response to viral infection can be summarized as follows: (1) modulation of the IFN signaling pathway and inflammatory factors; (2) regulation of immune cell development and function, such as B- and T cells. In the case of SARS-CoV-2 infection, what is noteworthy is that the virus can also manipulate host ncRNAs for its own replication and infection by acting as a sponge or magnet to absorb or hijack ncRNAs involved in the immune system.^101^

#### The IFN pathway

The deficiency of IFN immunity in the respiratory tract may result in the SARS-CoV-2 spread, causing pulmonary and systemic inflammation. Some host ncRNAs play critical roles in modulating the IFN pathway.

Let-7b-5p can target the phosphodiesterase 12 (PDE12) which seems to regulate the IFN response. Enhanced resistance to virus infection was found through inhibition of the PDE12, including encephalomyocarditis virus, human rhinovirus, and respiratory syncytial virus.^102^ However, with the aggravation of the COVID-19, let-7b-5p showed downregulation in severe COVID-19 cases,^48–50^ in line with the abnormal type I IFN response in several and critical patients, featured with the absence of both IFN-β and IFN-α production and activity,^103^ suggesting a role of let-7b-5p in the impaired IFN deficiency and reduced resistance to SARS-CoV-2 via modulating the PDE12.

There is a significant correlation between the differential expression of lncRNAs with protein-coding genes associated with the immune system. The lncRNA *LINC02384* can regulate IFN-γ expression to induce antiviral response and innate immune response.^104^ In addition, analysis of common miRNAs, circRNAs and mRNAs datasets revealed that hsa_circ_0080135 had multiple binding sites for 86 miRNAs, which were related to 15 mRNAs involved in cytokine storm including IL-1β, IL-7, IL-10, IL-12B, IL-13, IL-17A, IL-33, IFN-γ, C-C motif chemokine 2 (CCL2), C–X-C motif chemokine 6 (CXCL6), CXCL8, CXCL10, fibroblast growth factor (FGF2), FGF14 and macrophage inflammatory protein. On the other side, hsa_circ_0080135 also acted as ceRNA of miR-769-3p targeting IL-12B, IFN-γ, CXCL6 and CXCL8 to regulate cytokine storm.^105^ By targeting ncRNAs, plenty of dysregulated cytokines during SARS-CoV-2 infection were involved in the circRNA/lncRNA-miRNA-mRNA axis.

#### Inflammatory cascades

After SARS-CoV-2 infection, both pro-inflammatory and anti-inflammatory cytokines exhibit a dramatic elevation, and their expression and function can be mediated by host ncRNAs.^106^ One example is miR-106b-5p, which is a modulator of the lysine acetyltransferase 2B (KAT2B).^107^ Either pharmacological inhibition or knockdown of KAT2B resulted in decreased level of IL-10 in normal colonic epithelial cell line.^108^ In COVID-19 cases, KAT2B showed upregulation, while miR-106b-5p was downregulated, suggesting a possible routine that the decreased miR-106b-5p induced by SARS-CoV-2 results in high expression of KAT2B and promotes the IL-10 level.^50,52,107^

LncRNAs, particularly nuclear paraspeckle assembly transcript 1 (*NEAT1*) and metastasis-associated lung adenocarcinoma transcript 1 (*MALAT-1*), have been shown to play critical roles in the expression of pro-inflammatory cytokines. In patients infected with higher viral loads of SARS-CoV-2, a greater proportion of upregulated transcripts were represented by lncRNAs, which functionally correlated with lymphocyte activation and cytokine signaling.^109^ *NEAT1* shows high correlation with the cytokines^110^ and can serve as an immunoregulator on promoting the monocyte-macrophage differentiation.^111^ Further, knockdown of *NEAT1* in the human monocyte-macrophage cells inhibited the apoptosis and reduced the expression of cyclooxygenase-2 (COX-2) and several pro-inflammatory cytokines, such as the IL-6 and tumor necrosis factor α (TNF-α), possibly through targeting miR-342-3p.^112^ In the COVID-19 cases, *NEAT1* was upregulated,^36,50,113^ and consistently, miR-342-3p was downregulated,^43^ indicating that overexpressed *NEAT1* may facilitate the pro-inflammatory process through repressing miR-342-3p after SARS-CoV-2 infection.

CircRNAs also acts a pivotal function in the formation of pro-inflammatory cytokines. Compared with the healthy controls, hsa_circ_0000479 exhibited increased level in COVID-19 patients, along with the upregulation of retinoic acid-inducible gene I (RIG-I) and IL-6, and downregulation of miR-149-5p.^114^ Overexpressed hsa_circ_0000479 can indirectly stimulate the RIG-I through binding to miR-149-5p, and the activated RIG-I would trigger the expression of IL-6.^115,116^ Therefore, a hsa_circ_0000479 composed circRNA-miRNA-mRNA regulatory axis may serves a critical function in mediating the pro-inflammatory cytokines expression after SARS-CoV-2 infection.

#### T cells development and function

After almost all SARS-CoV-2 infections, T-cell responses can be detected. In a study recruiting 116 hospitalized COVID-19 patients with varying severity, mass cytometry of whole blood found decreased overall T cells and increased activated and cytotoxic CD8^+^ T cells in more severe cases, indicating a dysregulation of T-cell response in severe COVID-19 disease.^117^ The two upregulated DElncRNAs HIF1alpha-antisense RNA 1 (*HIF1A-AS-1)* and retinoid acid receptor-related orphan receptor alpha-antisense 7 (*RORA-AS-7)* were enriched in differentiation of T-helper cells, which can regulate T-cell differentiation, while the detailed underpinnings merit more investigations.^42^

### Impact on multiorgan deficits and long COVID

COVID-19 is recognized as a respiratory disease that causes significant pulmonary damage, but with disease progression, numerous extrapulmonary symptoms have been reported in patients, affecting various systems such as cardiovascular, neurological and endocrinological. There are various hypotheses about the pathogenesis of multiorgan failure in COVID-19, such as dysregulated immune response, viral toxicity, and throboinflammation.^68,118,119^ Of these, direct viral invasion-induced toxicity may be unique, due to organotropism of SARS-CoV-2 toward the respiratory tract, neurologic, myocardial, pharyngeal and gastrointestinal tissues along with wide expression of ACE2 and TMPRSS2 in the host body.^120,121^ COVID-19 progression is not limited to moderate or severe cases, as recent studies have demonstrated the persistence of a range of symptoms following acute infection, commonly referred to long COVID.^122^ Some hypotheses about the long COVID pathogenesis have been proposed, such as impaired autoimmunity, viral remnants, dysregulated dysbiosis, and tissue damage.^7^ However, the detailed mechanism that underlies long COVID remains unclear.

In light of the organ-specific functions exhibited by host ncRNAs, we elucidate their respective roles across different anatomical systems below, and the following findings suggest that the inflammatory signaling and tissue development both represent the predominant targets for host ncRNAs in regulating the progression of organ failures within the context of COVID-19.

#### Respiratory system

For COVID-19 cases, up to 20% will develop to a severe form, featured with the occurrence of COVID-19-associated ARDS, severe pneumonia, and pulmonary fibrosis.^123^ Furthermore, several investigations have reported persistent lung injury even after clearance of SARS-CoV-2. A meta-analysis examining chest computed tomography (CT) findings about 12 months post COVID-19 revealed that around 33% of patients still exhibited residual lung abnormalities on CT scans. These findings suggest that SARS-CoV-2 infection possibly leads to prolonged lung injury.^124^ Current perspectives on the pathogenesis of lung injury in COVID-19 primarily focus on direct viral damage and host immune response.^125^

As previously mentioned, dysregulated immune systems can trigger a cytokine storm that can damage alveolar structures, allowing the virus to invade vascular endothelial cells from the blood-air barrier. With the disease advances, endothelial dysfunction results in increased rigidity and susceptibility of pulmonary vessels., ultimately resulting in thrombosis and microvessels blockage in alveolar capillaries, potentially causing hypoxemia or pulmonary hypertension.^123^ The circulating miR-486-5p, which is decreased in COVID-19, can targets the OUT domain-containing protein 7B (*OTUD7B)* genes to regulate antiviral response and promotes acute lung injury.^88^ Several lncRNAs including *MALAT-1* and structural maintenance of chromosomes 2-antisense 1 (*SMC2-AS1)*, which separately regulates the IL-8, calpain-1 catalytic subunit 1 (CAPN1), Wnt, and TGF-β signaling pathway, are also essential to regulates lung repair and regeneration.^50,126^

#### Cardiac system

More than 7% COVID-19 patients experience myocardial injury from the infection, and over 25% of hospitalized cases showed an elevated level of troponin (a marker of cardiac dysfunction).^127–129^ Despite the mechanism underlying the cardiac injury after SARS-CoV-2 infection remains uncertain, direct viral damage received much attention. Many studies have showed that SARS-CoV-2 RNA can be observed in the heart from some COVID-19 cases.^120,130^ In addition, the cardiac myocyte apoptosis induced by cytokine storm and hypoxia-induced excessive intracellular calcium may also account for the cardiac injury in the context of COVID-19.^131^

The miR-208a and miR-499, two heart-muscle specific miRNAs, showed significant upregulation in the COVID-19 patients compared to the influenza-ARDS patients, possibly indicating chronic myocardial damage after SARS-CoV-2 infection.^46^ Otherwise, the miRNA miR-133a, which can regulate neutrophil counts and degranulation, plays important roles in inflammation-induced myocyte damage.^49^

#### Central nervous system

SARS-CoV-2 has been identified to significantly affect the central nervous system (CNS). Reports indicate that ~30% of hospitalized COVID-19 cases, 45% of severe cases, and 85% of patients with ARDS exhibit neurological symptoms.^132,133^

Current hypotheses aiming at the influence of SARS-CoV-2 on the CNS primarily focus on neuroinflammation and tissue damage, both of which involve host ncRNAs.^134^ Several ncRNAs show potential for modulating the immune process of neural cells. As we screened, an important lncRNA *NEAT1*, its elevation can regulate the inflammation of neurons and involve in the susceptibility to COVID-19 infection.^135,136^ Upregulated *NEAT1* was also reported in patients with ischemic stroke, and knockdown of *NEAT1* can alleviate the apoptosis and improve neuronal viability.^137^ This result suggests a possible role of *NEAT1* in the pathogenesis of stroke in COVID-19 cases. Moreover, some miRNAs, such as let-7c-5p, were reported to emerge as neuroprotective factors to inhibit microglia activation.^138^ Overexpression of let-7c-5p can reduce the infarction volume and improve the neurologic deficits. The SARS-CoV-2 has been observed in human brain vessels and can infect and damage neurons, indicating that the nervous system is vulnerable to attack by SARS-CoV-2.^139–142^

Psychiatric and neurological symptoms have been frequently reported in COVID-19 survivors for up to 12 months following infection, with the estimated prevalence of 19.7% and 18.7%, respectively.^9^ Sleep disturbances, depression, insomnia, anxiety symptoms, and cognitive impairment are prevalent in these individuals, placing a significant burden on their well-being.^143^ Besides, long COVID cases have reported experiencing “brain fog,” which is characterized by the feeling of being mentally slow or fuzzy.^144,145^ One potential mechanism by which ncRNAs may contribute to the development of neuropsychiatric sequelae is through modulation of aberrant neurotransmitter levels resulting from hyperinflammation. In individuals who recovered from COVID-19, miR-15a-5p was upregulated compared with that in healthy control, along with decreased serum soluble programmed cell death protein-1 (PD-1, a direct target of miR-15a-5p) and increased cytokines, including IL-1β, IL-1RA, and IL-8.^146^ The abnormal PD-1 signal may result in dysregulated T-cell functions, and inhibition of PD-1 can reduce the availability of tryptophan and tyrosine in the mice brain and repress the synthesis of serotonin and dopamine, leading to enhanced anxiety-like behaviors and fear response.^147^ These data suggest a function of the abnormal miR-15a-5p/PD-1 axis in the depression, anxiety or post-traumatic stress disorder symptoms in long COVID cases. Moreover, the function of miR-15a-5p in neuropsychiatric sequela of COVID-19 has more possibilities. Ataxin-7-like protein 3B (ATXN7L3B), a downstream target of miR-15a-5p, showed an involvement in human neurodevelopmental delay and ataxia,^148^ which possibly have a more dramatic effect on brain development in children who had been infected with SARS-CoV-2.

#### Peripheral nervous system

SARS-CoV-2 also induces a plethora of peripheral nervous system diseases both acutely and chronically. The common peripheral manifestations of COVID-19 include muscle pain, injury, fatigue and weakness. In COVID-19, skeletal muscle injury present in 19.3% of individuals who are severely ill and 4.8% of individuals in non-severe group.^133^ Fatigue and weakness have been commonly reported in individuals who recovered from COVID-19. Studies have showed that 32% of individuals continued to experience fatigue for 12 or more weeks after their initial COVID-19 diagnosis.^149^ Fatigue is characterized by an overwhelming feeling of tiredness or lack of energy, while weakness refers to a decrease in muscle strength.^150^ The skeletal muscles and other cells in muscles, including leukocytes, fibroblasts and endothelial cells, also express ACE2 receptors. Therefore, it suggests that skeletal muscles are susceptible to virus invasion and immune-mediated myopathies.^151^ The common gene interaction networks were shown between the long COVID and myalgic encephalomyelitis/chronic fatigue syndrome, involving 9 common genes and 102 miRNAs.^152^ In addition, a correlation was found between the downregulated let-7b-5p in convalescent individuals after SARS-CoV-2 infection and the master regulatory gene paired box protein 3 (*PAX3).*^42^ *PAX3* can mediate muscle function and protect muscle satellite cells from environmental stress.^153,154^ Further, it has been reported that upregulation of Pax3 in the myogenic differentiation antigen (MyoD^−/−^) myoblasts was accompanied with activated transcription of antiapoptotic factors B-cell lymphoma/leukemia-2 (Bcl-2) and Bcl-2-like protein-1 (Bcl-xL),^155^ while persistent expression of Pax3 would inhibit myogenic differentiation, indicating that approximate Pax3 degradation is critical for the progression of the myogenic program.^156^

## NcRNAs as biomarkers for COVID-19

Given the involvement of host ncRNAs in diverse processes including viral invasion and replication, immune response, multiorgan damage, and the occurrence of long COVID resulted from SARS-CoV-2 virus, distinct ncRNAs have emerged as potential biomarkers for each of these processes. Currently, ncRNAs have been utilized as promising biomarkers for kinds of diseases, mostly for multiple types of cancer,^10,11^ while the diagnostic utility of host ncRNAs may be limited in the context of COVID-19. Three diagnostic tests are commonly employed for COVID-19, encompassing molecular testing using nasopharyngeal or nasal swabs to detect viral RNA, antigen testing to identify viral proteins, and serology testing to detect host antibodies in response to infection, and the first two methods can be utilized for diagnosing acute infections.^157^

**Fig. 3:**
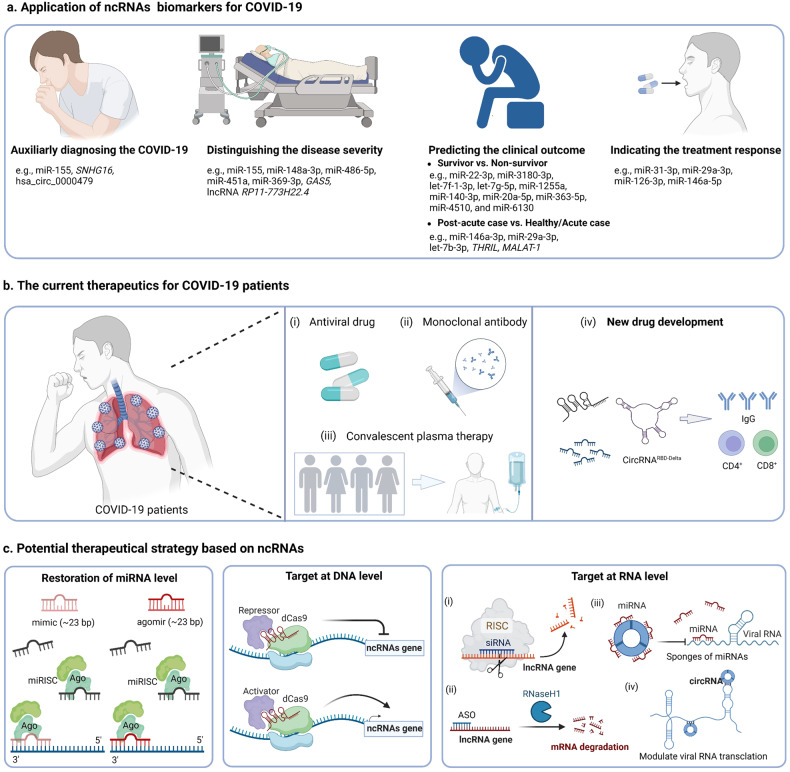
Summary of the clinical applications of ncRNAs-based biomarkers and therapeutic strategies against COVID-19**. a** Some host ncRNAs can be used as biomarkers in various clinical scenarios after SARS-CoV-2 infection, including auxiliary diagnosis of the COVID-19 (e.g., miR-155, *SNHG16*, hsa_circ_0000479), differentiation of the disease severity (e.g., miR-155, miR-148a-3p, miR-486-5p, miR-451a, miR-369-3p, *GAS5*, lncRNA *RP11-773H22.4*), distinguishing the survivors compared to non-survivors (e.g., a signature consisting of miR-22-3p, miR-3180-3p, let-7f-1-3p, let-7g-5p, miR-1255a, miR-140-3p, miR-20a-5p, miR-363-5p, miR-4510, and miR-6130), distinguishing the post-acute patients compared to healthy controls/acute patients (e.g., miR-146a-3p, miR-29a-3p, let-7b-3p, *THRIL, MALAT-1*), and indicating the treatment response (e.g., miR-31-3p, miR-29a-3p, miR-126-3p, miR-146a-5p). **b** The current therapeutics for COVID-19 patients, including (i) antiviral drugs, (ii) monoclonal antibody, (iii) convalescent plasma therapy and (iv) new drug development (ncRNAs-based strategy, e.g., circRNA^RBD-Delta^). **c** Potential therapeutical strategies based on ncRNAs. Restoration of miRNA level: the miRNA mimics and agomirs can be synthesized and delivered into the cells to increase the level of a target miRNA. Targeting ncRNAs at the DNA level: using CRISPRi and CRISPRa tools to transcriptionally inhibit or activate target ncRNA expression. Targeting ncRNAs at the RNA level: (i) The siRNA targets at ncRNAs in the RISC complex and initiate degradation of ncRNAs; (ii) ASOs can bind the target ncRNAs and induce its degradation by recruitment of Ribonuclease (RNaseH1); (iii) CircRNAs emerge as the sponges of miRNAs to restrain their bio-accessibility to mRNA; (iv) CircRNAs directly bind the viral mRNA to inhibit their propagation

Host ncRNAs may provide advantages in closely monitoring and evaluating the development of COVID-19 disease. According to WHO,^158^ severe COVID-19 patients are defined by any of: (1) oxygen saturation less than 90% on room air; (2) severe pneumonia; (3) signs of severe respiratory distress. The critical COVID-19 patients are defined by the criteria for ARDS, sepsis, septic shock, or other conditions that would normally require the provision of life-sustaining therapies such as mechanical ventilation or vasopressor therapy. Meanwhile, the WHO has also provided corresponding management and treatment recommendations for patients with different severity levels. During these processes, relying solely on clinical symptoms for disease progression assessment may lead to treatment delays, as molecular alterations in the body might have already occurred prior to symptom manifestation. Therefore, utilizing ncRNAs to assist the disease management can facilitate the convenience in monitoring the progression of COVID-19 and offer significant advantages in delivering timely treatment recommendations and measures. Additionally, compared to other molecular biomarkers, ncRNA possesses some features that make it a valuable tool in clinical use, including its tissue-specificity, cell-specificity, developmental stage-specificity, and stability.^159^ In the following section, we present a comprehensive overview of the current research on potential biomarker ncRNAs for COVID-19 diagnosis, stratification, prognostic evaluation, and treatment response (Fig. 3a).

### Diagnostic biomarkers for COVID-19

Some patients may persistently exhibit negative test results despite displaying clinical symptoms.^157^ Considering that the expression profiles of host miRNAs or lncRNAs are highly sensitive, with changes observed even between the acute phase (within 3 days) and later stages (approximately 7 days), and thus, host ncRNAs could serve as a valuable tool for confirming clinical diagnoses.^41,44,55^ For instance, the miR-155 molecule has been extensively recognized as a pivotal regulator of immune cells throughout evolution and serves a crucial role in the development of progressive inflammatory diseases.^41^ It exhibited a remarkably high area under the curve (AUC) value of 0.99 for COVID-19 diagnosis.^160^ Whether this differential expression of miR-155 is induced by SARS-CoV-2 infection or just due to the inflammatory response merits more investigations. However, an investigation reported that it was also upregulated of the miR-155 in the COVID-19 individuals compared to influenza-ARDS cases, showing a strong discrimination with an AUC of 1.00.^46^ More validations are worthy to identify whether miR-155 serves a specific function in the COVID-19 pathogenesis.

Several lncRNAs and circRNAs have also demonstrated promising outcomes. Among them, small nucleolar RNA host gene 16 (*SNHG16)* can activate the TGF pathway and participate in inflammatory cascades; its expression was found to be downregulated in COVID-19 cases compared to controls. Furthermore, *SNHG16* holds potential as a biomarker for distinguishing COVID-19 cases from healthy controls with an AUC of 0.67, sensitivity of 0.70, and specificity of 0.59.^161^ CircRNA has_circ_0000479 also showed upregulation in the COVID-19 patients, with a negative correlation with IL-6 expression.^114^

### Stratification biomarkers for COVID-19

Most individuals infected with COVID-19 typically experience mild (40%) or moderate (40%) manifestations of the disease. Approximately 15% progress to a severe stage necessitating oxygen support, while 5% develop critical illness characterized by complications likely respiratory failure, ARDS, sepsis and septic shock, thromboembolism, and/or multiorgan failure.^162^ Distinguishing between non-severe and severe COVID-19 patients is reliant on certain diagnostic examinations, including respiratory rate and chest CT. The availability of more accessible biomarkers for monitoring disease severity could facilitate prompt and appropriate treatment for patients. A selection of potential host miRNAs and lncRNAs were identified for discriminating COVID-19 severity (Table 2).

**Table 2:** Host miRNAs and lncRNAs with potential roles in discriminating COVID-19 and predicting clinical outcomes

Application	Host ncRNAs	Biological functions related with COVID-19 pathogenesis	Source	Expression	Sample size	AUC	Sensitivity	specificity	*P* value	Ref.
Discriminating COVID-19 patients with different severities										
Distinguishes between severe and moderate COVID-19 patients	miR-155	Regulating inflammatory-related proteins, immunomodulatory proteins, and tumor-supressor proteins	Plasma	Upregulation in severe cases	150	0.75	0.76	0.76	NA	^160^
	miR-200	Downregulating ACE2 expression, increasing angiotensin II level	Serum	Upregulation in severe cases	200	0.66	0.65	0.63	<0.0001	^167^
	GAS5	Regulating the miR-200/ACE2 axis involved in the occurrence of ARDS	Serum	Downregulation in severe cases	200	0.74	0.74	0.71	<0.0001	^167^
Distinguishes between severe and mild COVID-19 patients	*lncRNA RP11-773H22.4*	Regulating inflammation	Serum	Upregulation in severe cases	200	0.78	0.78	0.71	0.05	^32^
Distinguishes between ICU and ward COVID-19 patients	A signature consisting of miR-148a-3p, miR-486-5p, and miR-451a	miR-148a-3p targeting in the ORF1a, E, S, and M genes in the SARS-CoV viral genome; miR-486-5p promoting acute lung injury by inducing inflammation; miR-451a regulating cytokine and chemokine synthesis	Plasma	miR-148a-3p upregulation in ICU cases; miR-486-5p and miR-451a downregulation in ICU cases	79	0.89	NA	NA	NA	^88^
Distinguishing between COVID-19 patients with ARDS requiring MV and patients without MV	miR-369-3p	A dual role in both immune system regulation and viral performance	Serum	Downregulation in COVID-19 patients with ARDS requiring MV	20	0.72	NA	NA	0.05	^165^
Predicting COVID-19 clinical outcomes										
Predicting COVID-19-related death at 90 days	A signature including miR-22-3p, miR-3180-3p, let-7f-1-3p, let-7g-5p, miR-1255a, miR-140-3p, miR-20a-5p, miR-363-5p, miR-4510, and miR-6130	let-7g-5p, miR-363-5p, and miR-4510 targeting the SARS-CoV-2 genome; miR-140-3p targeting the serine protease TMRSS2 which process the viral invasion; miR-20a-5p targeting several proteins which may mediate SARS-CoV-2 induced cell death	Plasma	NA	96	0.97	0.92	0.93	NA	^43^
Differentiating ICU non-survivors from survivors	A signature including miR-192-5p and miR-323a-3p	miR-192-5p regulating cytokine and chemokine synthesis; miR-323a-3p inhibiting replication of the viral infection	Plasma	Downregulation in ICU non-survivors	36	0.80	NA	NA	NA	^88^
Differentiating COVID-19 non-survivors from patients who died due to other causes	miR-26a-5p	Involving in endothelial dysfunction and viral infection	Lung biopsies	Downregulation in COVID-19 cases	19	0.83	NA	NA	0.06	^40^
	miR-29b-3p	Involving in immune and adaptive response	Lung biopsies	Downregulation in COVID-19 cases	19	0.81	NA	NA	0.05	^40^
Discriminating post-acute COVID-19 patient										
Distinguishing post-acute COVID-19 patients from healthy control	miR-146a-3p	Positively correlated with dry cough, fever, and decreased smell	PBMCs	Upregulation in post-acute COVID-19 cases	33	0.98	NA	NA	<0.0001	^45^
	miR-29a-3p	Positively correlated with dry cough	PBMCs	Upregulation in post-acute COVID-19 cases	33	1	NA	NA	<0.0001	^45^
	let-7b-3p	Positively correlated with dry cough	PBMCs	Upregulation in post-acute COVID-19 cases	33	0.93	NA	NA	<0.0001	^45^
	miR-155-5p	Regulating inflammation and antiviral cellular defense	PBMCs	Upregulation in post-acute COVID-19 cases	40	0.83	NA	NA	<0.0001	^41^
Distinguishing acute COVID-19 patients from post-acute patients	miR-146a-3p	Positively correlated with dry cough, fever, and decreased smell	PBMCs	Upregulation in post-acute COVID-19 cases	18	0.80	NA	NA	0.001	^45^
	miR-29a-3p	Positively correlated with dry cough	PBMCs	Upregulation in post-acute COVID-19 cases	18	0.82	NA	NA	0.001	^45^
	THRIL	Controlling the expression of TNF-α signaling which regulates inflammation and immune response	PBMCs	Downregulation in post-acute COVID-19 cases	20	0.75	NA	NA	0.005	^41^
	MALAT-1	Controlling cytokine secretion in macrophages under inflammatory circumstances and promoting inflammatory activity by interacting with the NF-κB pathway	PBMCs	Downregulation in post-acute COVID-19 cases	20	0.72	NA	NA	0.021	^41^

*ARDS* associated acute respiratory distress syndrome, *GAS5* arrest-specific transcript 5, *MALAT-1* metastasis-associated lung adenocarcinoma transcript 1, *ORF* open-reading frame, *PB* peripheral blood, *PBMCs* peripheral blood mononuclear cells, *THRIL* TNF and HNRNPL-related immunoregulatory long non-coding RNA

MiR-155 plays a pivotal function in the regulation of inflammatory-related proteins and immunomodulatory proteins, exhibiting distinct expression patterns between either COVID-19 patients and healthy controls or severe and moderate cases.^160^ Haroun et al. identified an upregulation of plasma miR-155 in severe COVID-19 cases using RT-qPCR. Furthermore, they consistently observed a significant positive correlation between its expression level and clinical parameters likely chest CT findings, C-reactive protein (CRP), and ferritin levels. The AUC for miR-155 in distinguishing severe from moderate cases was 0.75, with a sensitivity and specificity of 0.76 each.^160^ Considering the above promising diagnostic implications,^45^ miR-155 holds more potential as an indicator for long-term monitoring of COVID-19 progression.

Certain miRNAs, including miR-148a-3p, miR-486-5p, and miR-451a, exhibit potential for distinguishing between COVID-19 patients in the intensive care unit (ICU) and those in general wards. As we have mentioned in the above section, miR-148a-3p can target various genes within the SARS-CoV2 genome (ORF1a, E, S, and M), and the other two miRNAs have been reported with dysregulated B and T lymphocytes, chronic inflammatory response, and acute lung injury.^163,164^ De Gonzalo et al. observed an upregulation of miR-148a-3p and downregulation of both miR-486-5p and miR-451a among ICU cases’ serum samples. The AUC value of a signature consisting of these three miRNAs for differentiating between ICU patients versus ward patients was 0.89, which was higher than other molecular biomarkers, such as leukocyte counts (AUC = 0.74), D-dimer (AUC = 0.87), or CRP (AUC = 0.72), indicating a value of miRNAs as biomarker for evaluating the COVID-19 development.

In addition to classifying disease severity, certain ncRNAs have the potential to predict adverse outcomes characterized by a rapid onset, as many miRNAs and lncRNAs showed differential expression in COVID-19 patients between the acute period (within 3 days) and later phage (7 days).^44,55^ Moreover, by comparing COVID-19 patients with ARDS requiring mechanical ventilation to those without mechanical ventilation, a significant downregulation of miR-369-3p was identified in the serum of patients needing mechanical ventilation. Furthermore, its AUC for discriminating between patients with and without mechanical ventilation was calculated as 0.72.^165^ Numerous studies have reported that miR-369-3p plays a dual role in both immune system regulation and viral performance; its downregulation can promote the production of inflammatory factors and it possesses a target site within the SARS-CoV-2 genome, indicating that the dysregulation of miR-369-3p following the onset of disease may facilitate and expedite the development of ARDS after SARS-CoV-2 infection.^165^

LncRNAs can also be utilized biomarkers for distinguishing the disease severity. For instance, lncRNA growth arrest-specific transcript 5 (*GAS5*) participated in promoting ACE2 expression by inhibiting miR-200.^166^ Accordingly, a contrasting expression pattern of *GAS5* and miR-200 was observed in the serum samples from 88 severe COVID-19 cases compared to 112 moderate cases, with a downregulation of *GAS5* and an upregulation of miR-200.^167^ Notably, *GAS5* exhibited superior discriminatory performance between severe and moderate patients, as evidenced by an AUC of 0.74 (sensitivity=0.74, specificity=0.71), while miR-200 demonstrated an AUC of 0.66 (sensitivity=0.65, specificity=0.63). LncRNA *RP11-773H22.4* also showed potential for differentiating severe and mild COVID-19 patients; its serum expression was increased in the severe patients compared to mild ones, which could cause downregulated miR-4257 and subsequently upregulated IL-11 receptor subunit alpha (IL-11RA) mRNA thereby promoting inflammation.^32^ The AUC for lncRNA *RP11-773H22.4* in this cohort was 0.78, with a sensitivity of 0.78 and specificity of 0.71. Additionally, multivariate analysis revealed that lncRNA *RP11-773H22.4* was an independent factor besides serum ferritin level and CT findings, demonstrating its promising role as a predictor for COVID-19 severity.

### Prognostic biomarkers for COVID-19

#### Survivors vs. non-survivors

As the disease progresses, some patients may succumb to it. A systematic analysis of COVID-19-related mortality from 2020 to 2021 revealed a global all-age excess mortality rate of 120.3 deaths (113.1–129.3) per 100,000 population due to COVID-19.^168^ Therefore, it is imperative to investigate early markers for the COVID-19 clinical outcomes forecast, and certain miRNAs exhibit potential in this domain. Collecting the plasma sample from COVID-19 patients upon hospital admission or within the first few days after hospitalization but before treatment, 77 upregulated miRNAs and 60 downregulated miRNAs in severe cases were identified.^43^ Furthermore, a mortality predictive model consisting of ten miRNAs (miR-22-3p, miR-3180-3p, let-7f-1-3p, let-7g-5p, miR-1255a, miR-140-3p, miR-20a-5p, miR-363-5p, miR-4510, and miR-6130) was constructed and confirmed to have better predictive power than the basic model that only considered age and gender (AUC: 0.97 vs. 0.88), along with over 90% sensitivity and specificity. Similarly, miR-192-5p and miR-323a-3p showed downregulation in ICU COVID-19 non-survivors compared with the survivors, and a signature composed of them can be utilized for discriminating the non-survivors from survivors with an AUC of 0.8. Among the above host miRNAs, some of them (e.g., let-7g-tp, miR-363-5p, and miR-4510) can target the SARS-CoV-2 genome, and miR-323a-3p may play an inhibitory role in viral replication; miR-140-3p can target the serine protease TMPRSS2, showing a role in regulating the viral invasion; miR-20a-5p and miR-192-5p can participant in the host response, such as cell death and cytokine synthesis.^43,88^ The dysregulated expression of these host miRNAs indicates a distinction in viral invasion and replication as well as subsequent host response between COVID-19 non-survivors and survivors, potentially manifesting earlier than clinical symptoms. Therefore, these miRNAs may serve as both prognostic biomarkers for mortality risk prediction and therapeutic targets for mitigating COVID-19 progression.

#### Post-acute patients vs. healthy control/acute patients

Following the acute phase, a considerable number of patients may encounter persistent manifestations subsequent to their initial symptomatic SARS-CoV-2 infection, commonly named as long COVID. The host ncRNAs may be involved in this process, as evidenced either by the identification of numerous host DEmiRNAs and DElncRNAs during the recovery stage of COVID-19 or the possible functions in the persistent organ failures.^42^ In line with these findings, a study comparing host miRNAs in PBMCs between the post-acute phase COVID-19 patients (4–5 weeks after the acute phase) and the healthy controls revealed upregulation of three miRNAs (miR-146a-3p, miR-29a-3p, let-7b-3p) among post-acute cases.^45^ All three exhibited an AUC value above 0.9 for discriminating between post-acute COVID-19 cases and healthy controls. Furthermore, the expression levels of miR-146a-3p and miR-29a-3p were found to be higher in PBMCs during the post-acute stage compared to the acute phase, indicating a progressive increase in their expressions throughout COVID-19 development. Some findings indicate that miR-146a possibly exerts a protective effect on the virus by suppressing signal transducer and activator of transcription 1 (STAT1) protein, thereby impeding SARS-CoV-2 replication and evading antiviral response.^169,170^ Moreover, previous studies demonstrated that miR-146a-3p negatively regulated the Sirtuin-1/noncanonical nuclear factor-κB (NF-κB) axis to contribute to acute lung injury.^45,171^ This is consistent with that miR-146a has been shown to target 21 differentially expressed genes (DEGs) in lung tissues of COVID-19 patients.^172^ Accordingly, a positive correlation was observed between miR-146a-3p expression and manifestations such as fever, and coughing in COVID-19 cases. Consequently, stepwise upregulation of miR-146a-3p may play a role in persistent post-acute phase symptoms or even long COVID through various cascades, indicating its potential for closely monitoring COVID-19 development. A positive correlation was also observed between the expression of miR-29a-3p and dry cough in COVID-19 cases.^45^ However, further investigations are required to determine whether this miRNA exerts an antiviral or pro-viral function in SARS-CoV-2 pathogenesis, as some findings have shown downregulation of miR-29a-3p in the plasma of ARDS patients and administration of miR-29a-3p agomir can inhibit the expression of inflammatory factors in the lung.^173^

Certain lncRNAs, such as TNF-α and heterogeneous nuclear ribonucleoprotein L (*THRIL)* and *MALAT-1*, exhibited differential expression between post-acute and acute COVID-19 patients. *THRIL* can modulate TNF-α expression by interacting with heterogeneous nuclear ribonucleoprotein L, promoting inflammation and immune response.^174^ Similarly, *MALAT-1* can regulate cytokine secretion and contribute to inflammatory activity through targeting the NF-κB pathway.^175^ The expressions of these lncRNAs in PBMCs were significantly decreased during the post-acute phase (6–7 weeks after the acute phase), with AUC values for discriminating the post-acute cases from acute cases of 0.75 for *THRIL* and 0.72 for *MALAT-1*, respectively. Further, a positive correlation was identified between dry cough and *THRIL* expression, while fever and skeletal pain showed a positive correlation with *MALAT-1* expression, indicating possible involvement of host lncRNAs in persistent manifestations.^41^

### Predictive biomarkers for COVID-19 treatment response

In addition to monitoring and predicting the development of COVID-19, certain host miRNAs exhibit specific expression patterns in response to COVID-19 treatment. Notably, hospitalized patients with varying disease severity demonstrated significant downregulation of miR-31-3p, miR-29a-3p, and miR-126-3p levels. However, in patients treated with remdesivir and favipiravir during hospitalization, the expression of these three miRNAs returned to baseline levels in treatment-responsive patients compared to non-responsive individuals.^176^ Considering that miR-29a-3p also exhibited increased expression during the recovery stage compared to the acute phase, it can be speculated that this particular miRNA possibly acts as an marker of COVID-19 improvement and possibly play a pro-viral function. Another study analyzing serum samples from COVID-19 cases with multifocal interstitial pneumonia who received a single-dose intravenous infusion of tocilizumab—an anti-IL-6 receptor drug—revealed a marked increase in serum levels of miR-146a-5p among treatment-responsive patients. This finding is in line with its downregulation observed in COVID-19 patients in comparison with the healthy.^50,177^ These host miRNAs exhibit potential as predictive markers for personalized treatment response, thereby aiding in enhancing the efficacy of COVID-19 therapies. Further investigations are worthy to explore whether there exist host lncRNAs or circRNAs with analogous functionalities.

### Demographic factors influencing the expression of host ncRNAs

The severity and mortality of COVID-19, including long COVID, are influenced by various factors such as age, sex, and pre-existing comorbidities. A number of studies have shown that elderly individuals or males are frequently associated with worse COVID-19 prognosis.^178–180^ In turn, these demographic variables may also impact the host ncRNAs expression. Comparing COVID-19 patients in different conditions can aids in identifying DEncRNAs as potential biomarkers for targeted populations. However, the comprehensive impact of these factors on ncRNAs in COVID-19 patients remains inadequately documented. Current research has been limited to detecting changes in a specific type of miRNA using PCR methodology, and whether these differences extend to lncRNAs and circRNAs remains largely unexplored.

Some miRNAs exhibited an association with age in the context of COVID-19. The miR-10b (a miRNA regulating the cytokines) showed a negative correlation with the age of COVID-19 cases, along with a downregulation in COVID-19 patients compared with age-matched healthy controls, indicating that a greater decrease of miR-10b per age may be associated with the higher inflammation in the older COVID-19 patients.^30^ In addition, is there any differential expression of miRNA in COVID-19 patients across different age groups? Evidence from high-throughput methods remains limited, but a study utilizing RT-qPCR demonstrated that miR-200c-3p was upregulated in saliva samples of COVID-19 cases over the age of 42 compared to those under 42 years old.^181^ Combined with the finding that miR-200c-3p showed higher expression in severe COVID-19 cases *vs*. healthy control, it suggests that some miRNAs in older patients may contribute to increased inflammation and cytokine storm, thereby exacerbating disease severity and mortality.^52,181^

Sex and comorbidities can also affect the expression of host miRNAs in the context of COVID-19. Comparing with female COVID-19 cases, miR-10b was downregulated in the male ones who more possibly experienced frequent infection, poor clinical outcomes, and higher mortality.^179,182^ Besides, pre-existing comorbidities may modulate the severity of COVID-19 via miRNAs, due to a preliminary result that the expression of miR-200c-3p was independently associated with COVID-19 cases with hypertension.^181^

The overall findings of these investigations suggest a correlation between changes in the expression of certain miRNAs and age, gender or comorbidity in COVID-19 patients, potentially shedding light on the more severe symptoms observed in older or male patients and those with comorbidities. However, it is important to note that further investigations with larger sample sizes and advanced sequencing or microarray techniques are needed to determine whether these results specifically stem from the SARS-CoV-2 infection and whether can generalize to lncRNAs and circRNA.

## NcRNAs-based therapeutics for COVID-19

The emergence of specific therapies designed to modulate ncRNAs has opened up new possibilities for their use as therapeutic targets. Such strategies typically involve interventions that target the transcriptional activation or inhibition of ncRNA expression loci at either the RNA or DNA level. Examples of these interventions include the use of mimics, agomirs, clustered regularly interspaced short palindromic repeats (CRISPR)/CRISPR-associated protein 9 (Cas9) tools, antisense oligonucleotides (ASOs), and RNAi knockdown.^11^ The advancements made in gene editing techniques have resulted in an increase in the number of preclinical and clinical investigations which have explored the potential use of ncRNA candidates for treating a variety of diseases, such as liver cancer,^183^ viral hepatitis C,^184^ cardiovascular disease,^185^ and Alzheimer’s disease.^186^ Given this promising trend and important functions in the pathogenesis of COVID-19, ncRNAs may also represent a viable therapeutic approach for treating COVID-19, including cases of long COVID.

### Potential therapeutic targets of ncRNAs

One potential routine is the intervention of virus infection. Specifically, miR-150-5p shows great therapeutic values for the treatment of such infection. Increasing the expression level of miR-150-5p through the utilization of mimics has been recognized to attenuate SARS-CoV-2 infection in vitro, while inhibition of miR-150-5p can reverse this effect.^187^ Other ncRNAs, including miR-106-5p, let-7b-5p, and *NEAT1*, have also emerged as potential therapeutic targets due to their contributions to immune response and cellular development.

Repairment of immune response is the paramount treatment for COVID-19. NcRNAs-based therapeutics that target specific immune factors nucleotide sequences may mitigate inflammatory and cytokine storms, and ameliorate the immune response to SARS-CoV-2 infection.

NcRNAs may also act as targets for treating the neuropsychiatric symptoms or sequela following SARS-CoV-2 infection, with miRNAs such as miR-15a-5p and let-7 family playing essential roles as regulators of brain development in association with ataxia-associated genes such as *ATXN1*, *ATXN1L*, and *ATXN7L3B*.

### Promising therapies modulating the host ncRNAs

Thus far, a variety of therapeutics have been employed in clinical settings for treating COVID-19, which can be broadly categorized into three categories (Fig. 3b): drug repurposing, monoclonal antibody and convalescent plasma therapy. Several drug/vaccines have been successfully developed and used in COVID-19 patients, such as antiviral drug (remdesivir), hydroxychloroquine, combination of two anti-human immunodeficiency virus (HIV) drugs (lopinavir and ritonavir), glucocorticoids (dexamethasone) and monoclonal antibodies (REGEN-COV, tocilizumab, sotrovimad, regdanvimad and combination of bamlanivimab and etesevimab).^188–192^ Despite these successes, small-molecule inhibitors and vaccines are limited in their ability to target “undruggable” portions of the genome. NcRNAs, on the other hand, offer promising targets for therapeutic intervention, as they can regulate genes and affect viral replication and infection in a direct way. Recently, Qu et al.^193^ reported that circRNA^RBD-Omicron^ can induce more effective neutralizing antibodies and immune responses against SARS-CoV-2 variants than mRNA vaccines, indicating that ncRNA-based therapies hold significant potential for future applications.

MiRNA mimics and agomir are widely-used methods to increase the levels of miRNAs which have been downregulated in disease.^194–196^ MiRNA mimics are designed to have the same sequence as endogenous mature miRNAs, and can increase the levels of mature miRNAs and reorganize their targets (Fig. 3c).^197,198^ For instance, miR-219 has been recognized as a pivotal function in regulating the oligodendrocyte development, myelination, and remyelination.^199^ In a demyelinating model induced by Theiler’s murine encephalomyelitis virus, intranasal administration of miR-219 mimics before disease onset markedly improved the disease severity, along with reduction of pro-inflammatory cytokine levels and viral RNA replication.^200^ This result highlights the potential of host miRNA mimics for the treatment of viral diseases. Moreover, the ease with which miRNA mimics can be synthesized in commercial laboratories increases their availability and accessibility for clinical use.^185^

In a broader context, therapeutic interventions based on ncRNAs can be classified into two distinct categories: those that modulate transcription at the DNA level, and those that modulate transcription at the RNA level (Fig. 3c). To date, DNA genome-editing tools, such as CRISPR-interference and CRISPR-activation showed exciting efficiency to inhibit or activate ncRNAs expression. In this method, the mutant form of Cas9 is fused with transcriptional repressors or activators of the promoter of specific ncRNAs.^201,202^ The two main strategies of modulation RNA expression are ASOs and RNA-mediated interference (RNAi) which can inhibit the ncRNAs. The high affinity with the cell membrane and great transfection efficiency making ASOs and RNAi as promising ncRNAs-based inhibitory therapies.^203,204^ On the other side, circRNAs can sequester virus-associated miRNAs and restrain their bio-accessibility to mRNA, or circRNAs directly target the conserved regions of viral RNA to suppress its propagation.^205,206^ Thus, it is crucial to fully explore the potential possibility of ncRNAs-based therapeutics for COVID-19 and subsequent disease states.

## Conclusion and perspective

This review highlights the significance of host miRNAs, lncRNAs, and circRNAs in the pathogenesis of SARS-CoV-2, providing evidence for the potential clinical value of ncRNAs in the stratification, prediction, and treatment of COVID-19, including long COVID.

Increasing research has demonstrated that viral infections can induce widespread changes in host ncRNAs, which in turn can impact virus invasion and pathogenesis. Recent findings have revealed that the interaction between host miRNAs and RNA viruses can be either direct or indirect.^207^ In the indirect pathway, viral RNA is recognized by pattern-recognition receptors and Toll-like receptors, leading to IFN signaling cascade activation, which suppress viral replication. These processes may further alter miRNA expression levels with pro-viral or antiviral effects. In the direct pathway, host miRNAs directly bind to various regions of the viral genome such as 5’ UTR, 3’ UTR, or coding regions on different types of RNA viruses like Eastern equine encephalitis virus, primate foamy virus 1, HIV, influenza, Hepatitis C virus, as well as SARS-CoV-2. This direct interaction can result in the inhibition of viral genome translation to suppress viral replication or stabilization of virus RNA to promote replication. Additionally, altered miRNAs may be involved in host immune response and contribute to viral pathogenesis. Similarly, lncRNAs exhibit pleiotropic functions in modulating the pathogenesis of viruses.^208^ Commonly, lncRNAs regulate viral pathogenesis through several mechanisms, such as modulation of cytoplasmic RNA receptors involved in viral recognition, regulation of IFN genes and IFN-stimulated genes expression leading to either anti- or pro-viral replication properties, and direct modulation of IFN production by binding to the IFN promoter region. While there is a paucity of information about the circRNAs, some studies have highlighted their significance in modulating viral pathogenesis. The primary mechanism by which circRNAs function is through acting as miRNA sponges to influence various processes, including viral replication (e.g., SARS-CoV-1, MER-COV), immune response and inflammation.^209^ The existence of specific relationships between host ncRNAs and SARS-CoV-2 infection is an intriguing question. However, current research primarily focuses on investigating the dysregulated landscape of host ncRNAs following SARS-CoV-2 infection, with limited wet-lab experiments deciphering the underlying mechanisms behind this relationship. Further studies are warranted to explore their functions and relationships in order to facilitate our understanding of COVID-19 pathogenesis.

**Fig. 4:**
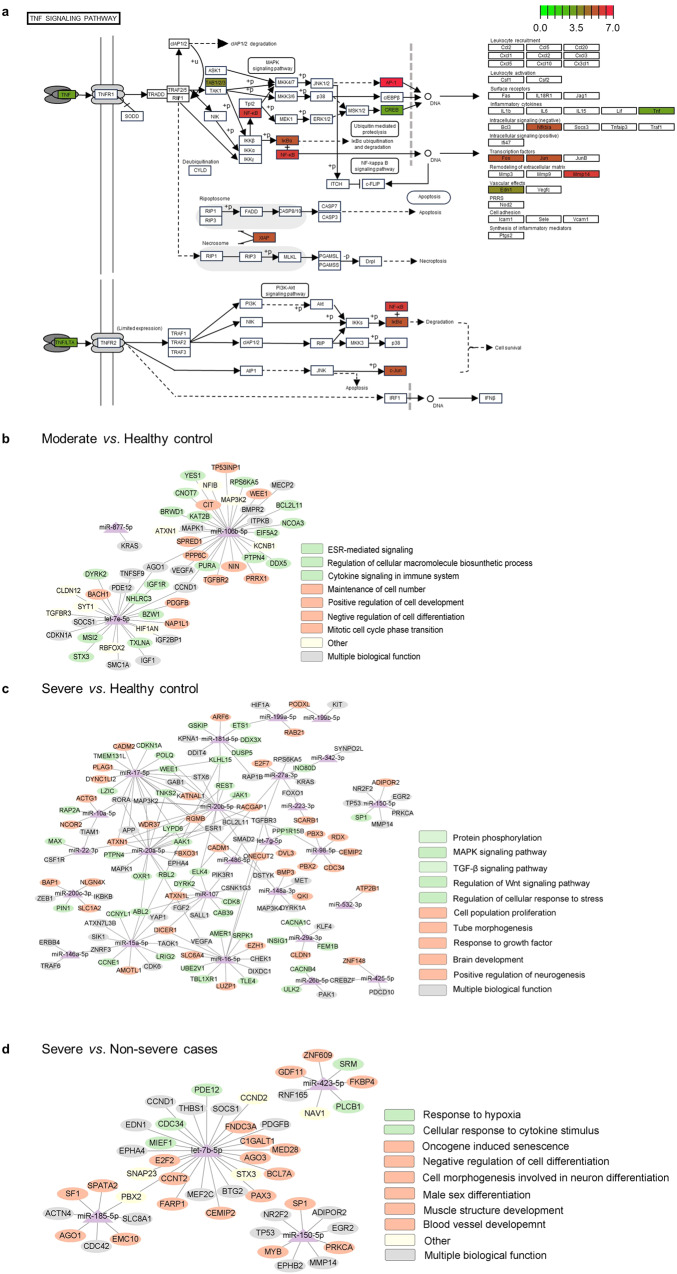
Gene function network analysis of some essential dysregulated miRNAs in COVID-19 cases. **a** Pathway showing the targets involved in the MAPK and TNF signaling pathway. The color indicates relevant reported times of a gene. **b** The miRNA-mRNA network in the group of COVID-19 moderate cases *vs*. healthy controls. **c** The miRNA-mRNA network in the group of COVID-19 severe cases *vs*. healthy controls. **d** The miRNA-mRNA network in the group of COVID-19 severe cases *vs*. non-severe cases. Different color represents relevant biological function. Green ovals represent immune response related pathways; Orange ovals represent multiorgan deficits related pathways; Purple triangles represent miRNAs

Based on the aforementioned findings, a plethora of host ncRNA, particularly miRNAs, have been reorganized as pivotal regulators in modulating pathogenesis to COVID-19. In order to further providing some directions for future studies, we took the miRNAs discussed in the section “SARS-CoV-2 associated ncRNAs” (Supplementary Tables S1–3) as input search, then performed a bioinformatic analysis of the target genes of the miRNA, finding that the immune response and organ deficits, even long COVID, may be promising routines for future investigation. As shown in Fig. 4a, COVID-19-related DEmiRNAs mostly enriched on the TNF pathway with the activation of NF-κB signaling. The aforementioned pathways exhibit a strong association with genes encoding inflammatory factors, including TNF, NF-κB, and inhibitor κB (IκB). In addition, some signaling pathway are highly linked with transcription genes, including transcription factor Jun (JUN), protein c-Fos (FOS), activator protein-1 (AP-1), and cAMP response element-binding protein (CREB). Several genes like *HIF1A* can interact with JUN and promote the regulatory effect of T cells to enhance virus clearance. This effect can be regulated by the DElncRNAs *HIF1A-AS-1*. The activation of AP-1 is required to interact with other genes like *RORA*, which is regulated by DElncRNA *RORA-AS-7*. The AP-1-associated pathway plays an essential function in controlling T-cell differentiation. Besides, several miRNAs show association with the development of some tissue or organs in the host after SARS-COV-2 infection. We found some miRNAs may play as regulatory factors involved in blood vessel development, which potentially result in the development of cardiovascular disease. Our bioinformatical results also indicate that some DEmiRNAs may regulate the brain development, neural differentiation, and neurogenesis via the interaction with key genes, including *DICER1, ATXN1, ATXN1L,* and syntaxin-6 *(STX6)* (Supplementary Fig. S1 and Fig. 4b–d), which potentially contribute to the development of cerebrovascular disease and neurodevelopmental diseases. The future requires further wet-lab experiments to validate their functionality in these processes, surpassing the confines of bioinformatics analysis.

To date, SARS-CoV-2 remains a persistently menacing pathogen to human beings in the foreseeable future. With the emergence of numerous variants of SARS-CoV-2, it is still unclear whether and how the evolutionary trajectories of coronaviruses impact the human genome. Sex can serve as a valuable lens for comprehending this inquiry, as compared to female, significantly disease severity and mortality have been found in male COVID-19 patients.^210^ Accordingly, elevated expression of virus entry factors, ACE2 and TMPRSS2, was observed in host Sertoli cells and germ cells, indicating a greater impact on males with reproductive disorders in COVID-19.^211^ Interestingly, our enrichment analysis results of the DEmiRNAs, which also have been reported before, showed that male sex differentiation with master regulatory gene katanin p60 ATPase-containing subunit A-like 1 (*KATNAL1)* is highly enriched in severe compared to non-severe cases. From a macroscopic perspective, the reproductive disorders following infection may drive some evolutionary adaptations within the human. The ncRNAs derived from the human genome, which are associated with immune response and pathological changes caused by SARS-CoV-2, could serve as evolutionary indicators under substantial selection pressure. In addition to elucidate viral diversity and disease severity, these host ncRNA indicators may also provide insights into the evolutionary trajectories and protection persistence after COVID-19. Further investigation and comparison over an extended temporal scale are imperative to comprehend the potential long-term impact of this selective pressure.

Many gaps are worthy of further exploring in the future. First, there remains a dearth of genome-wide screening of ncRNAs, especially the lncRNAs and circRNAs, expression in individuals of all ages, ranging from children to the elderly, who have been infected with SARS-CoV-2. The use of high-throughput sequencing would be advantageous in uncovering the expression profile of ncRNAs in COVID-19 cases, as well as identifying potential biomarkers and therapeutic targets. Second, multiple variants of SARS-CoV-2 have emerged. Distinct virus subtypes lead to varying symptoms, but little research has examined whether different ncRNA expression is induced.^212^ Exploring the expression profile of ncRNAs induced by different variants can improve our understanding of their influences. Third, the increasing burden of long COVID and reinfection have resulted in significant challenges, with the pathogenesis and treatments remaining unclear.^213–215^ Although some hypotheses have been proposed regarding the host ncRNAs in long COVID or reinfection, direct investigations about the functions are lacking. Thus, conducting comprehensive transcriptomic screening and wet-lab experiments of individuals with long COVID or reinfection necessitate further study.

NcRNAs hold significant therapeutic potential for patients. However, challenges such as off-target effects, specificity, and toxicity issues in drug design and delivery systems hinder their clinical translation. With the advancement of sequencing technologies and detection methods, more ncRNAs will emerge from the genome’s dark matter to pave the way for successful translational applications in COVID-19 patients and other human diseases.

## Supplementary information

Supplementary_Materials
