# Supplementary material for: Non-coding RNAs expression in SARS-CoV-2 infection: Pathogenesis, clinical significance and therapeutic targets
Source: Signal Transduct Target Ther. 2023 Dec 6;8:441. doi: 10.1038/s41392-023-01669-0 (PMC10700414; doi:10.1038/s41392-023-01669-0)
Supplement: Supplementary file 1 — Supplementary_Materials [file 41392_2023_1669_MOESM1_ESM.docx]

Supplementary Materials for

Non-coding RNAs expression in SARS-CoV-2 infection: pathogenesis, clinical significance and therapeutic targets

Xiaoxing Liu, Wandi Xiong, Maosen Ye, Tangsheng Lu, Kai Yuan, Suhua Chang, Ying Han, Yongxiang Wang, Lin Lu, Yanping Bao

Correspondence to: baoyp@bjmu.edu.cn, linlu@bjmu.edu.cn, wang-yongxiang@hotmail.com

**This PDF file includes:**

Method

Supplementary Figure S1

Tables S1 to S4

**METHODS**

**Functional enrichment analysis**

In the present study, we selected the target miRNAs in three groups, including moderate COVID-19 cases vs. health controls, severe cases vs. healthy controls, and severe cases vs. non-severe cases. Four databases, including miRDB, TargetScan, miRTarBase and miRWalk were applied to predict the interaction between miRNAs-gene target network. We used Metascape (http://metascape.org) to analyze functional enrichment analysis of the target genes of miRNAs. Next, we constructed miRNA-mRNA network using Cytoscape 3.9.1 software, which visualized the key nodes and intrinsic links among differentially expressed the target miRNAs. Values of p < 0.05 were statistically significant.

**Pathway analysis and associated mechanisms**

The KEGG were used to analyze the available pathway of target genes of comprehensive review of miRNAs. To visualize the regulatory relationships in the miRNA-mRNA network, we downloaded clusterProfiler and constructed an integrated functional network and signal pathways of these genes.

**SUPPLEMENTARY FIGURE LEGEND**

**Fig. S1 Functional enrichment analysis results.** The top 20 enriched biological functions (p < 0.05) were associated with miRNAs that were selected for moderate COVID-19 cases vs. healthy controls (a), severe COVID-19 cases vs. healthy controls (b), and severe cases vs. non-severe cases (c). The GO enriched terms are colored by p-value.


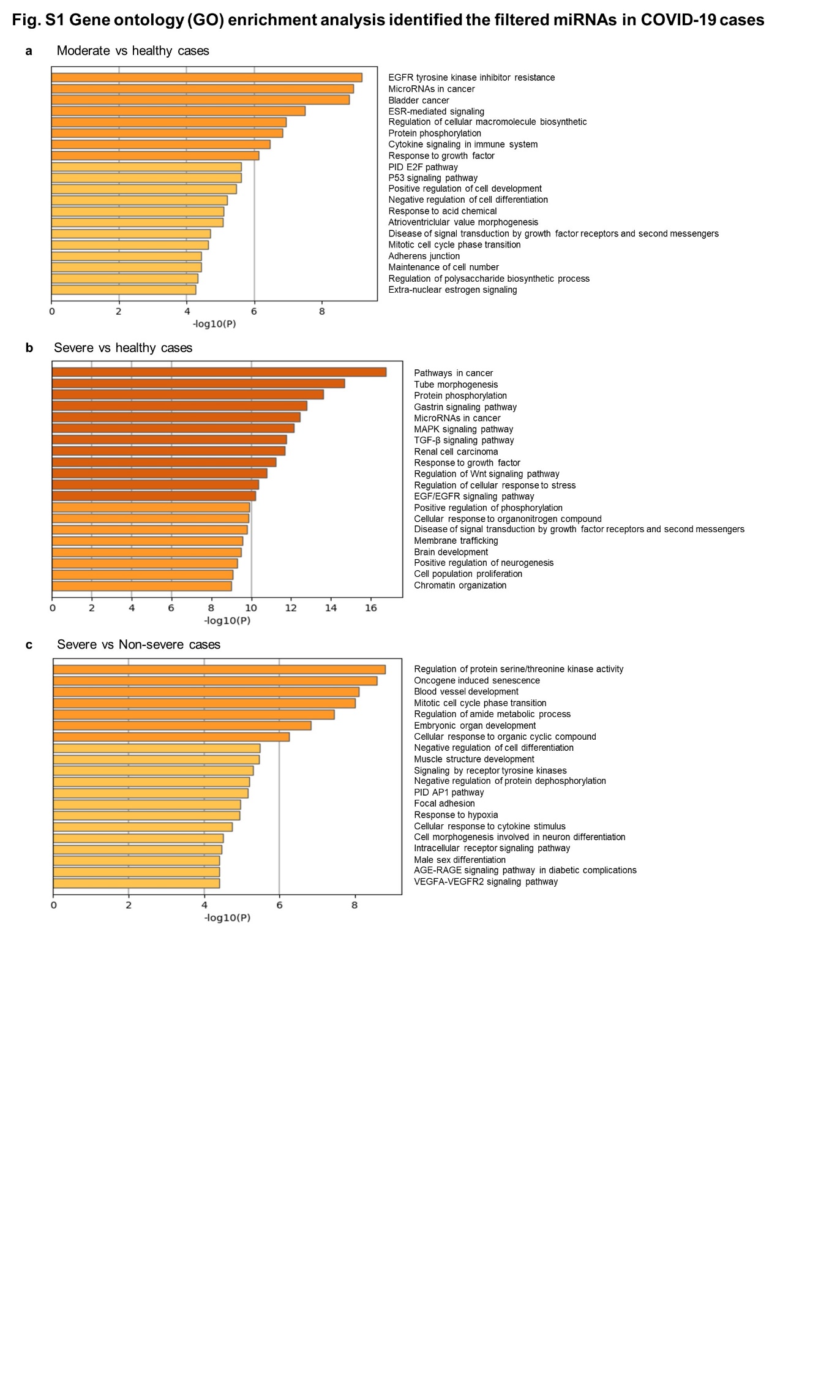


Table S1.

**Dysregulated miRNAs in moderate COVID-19 cases which used for bioinformatic analysis**

| **Moderate COVID-19 cases vs Healthy control** | | | |
| --- | --- | --- | --- |
| **Number** | **miRNA** | **Tissue type** | **Authors and years** |

| 1 | miR-197-3p | Plasma | Farr et al., 2021^1^ |
| --- | --- | --- | --- |
|  |  | Red blood cell-depleted whole blood | Tang et al., 2020^2^ |
| 2 | miR-345-5p | Plasma | Farr et al., 2021^1^ |
|  |  | Red blood cell-depleted whole blood | Tang et al., 2020^2^ |
| 3 | miR-339-3p | Plasma | Farr et al., 2021^1^ |
|  |  | Red blood cell-depleted whole blood | Tang et al., 2020^2^ |
| 4 | miR-21-3p | Plasma | Farr et al., 2021^1^ |
|  |  | Red blood cell-depleted whole blood | Tang et al., 2020^2^ |
| 5 | miR-1255b-5p | Plasma | Farr et al., 2021^1^ |
|  |  | Red blood cell-depleted whole blood | Tang et al., 2020^2^ |
| 6 | let-7a-3p | Plasma | Farr et al., 2021^1^ |
|  |  | Red blood cell-depleted whole blood | Tang et al., 2020^2^ |
| 7 | miR-7-1-3p | Plasma | Farr et al., 2021^1^ |
|  |  | Red blood cell-depleted whole blood | Tang et al., 2020^2^ |
| 8 | miR-106b-5p | Plasma | Farr et al., 2021^1^ |
|  |  | Red blood cell-depleted whole blood | Tang et al., 2020^2^ |
| 9 | miR-651-5p | Plasma | Farr et al., 2021^1^ |
|  |  | Red blood cell-depleted whole blood | Tang et al., 2020^2^ |

| 10 | let-7e-5p | Plasma | Farr et al., 2021^1^ |
| --- | --- | --- | --- |
|  |  | Red blood cell-depleted whole blood | Tang et al., 2020^2^ |
| 11 | miR-1273h-3p | Plasma | Farr et al., 2021^1^ |
|  |  | Red blood cell-depleted whole blood | Tang et al., 2020^2^ |
| 12 | miR-23a-5p | Plasma | Farr et al., 2021^1^ |
|  |  | Red blood cell-depleted whole blood | Tang et al., 2020^2^ |
| 13 | miR-450b-5p | Plasma | Farr et al., 2021^1^ |
|  |  | Red blood cell-depleted whole blood | Tang et al., 2020^2^ |
| 14 | miR-576-5p | Plasma | Farr et al., 2021^1^ |
|  |  | Red blood cell-depleted whole blood | Tang et al., 2020^2^ |
| 15 | miR-766-3p | Plasma | Farr et al., 2021^1^ |
|  |  | Red blood cell-depleted whole blood | Tang et al., 2020^2^ |
| 16 | miR-877-5p | Plasma | Farr et al., 2021^1^ |
|  |  | Red blood cell-depleted whole blood | Tang et al., 2020^2^ |

Table S2.

**Dysregulated miRNAs in severe COVID-19 cases which used for bioinformatic analysis**

| **Severe COVID-19 cases vs Healthy control** | | | |
| --- | --- | --- | --- |
| **Number** | **miRNA** | **Tissue type** | **Authors and years** |

| 1 | miR-150-5p | Plasma | Akula et al., 2022^3^ |
| --- | --- | --- | --- |
|  |  | Plasma | Farr et al., 2021^1^ |
|  |  | Plasma | Gutmann et al., 2022^4^ |
| 2 | miR-4433b-5p | Plasma | Farr et al., 2021^1^ |
|  |  | Plasma | Gutmann et al., 2022^4^ |
| 3 | miR-1246 | Plasma | Gutmann et al., 2022^4^ |
|  |  | Red blood cell-depleted whole blood | Tang et al., 2020^2^ |
| 4 | miR-197-3p | Plasma | Gutmann et al., 2022^4^ |
|  |  | Red blood cell-depleted whole blood | Tang et al., 2020^2^ |
| 5 | miR-532-3p | Plasma | Gutmann et al., 2022^4^ |
|  |  | Red blood cell-depleted whole blood | Tang et al., 2020^2^ |
| 6 | miR-140-3p | Plasma | Gutmann et al., 2022^4^ |
|  |  | Red blood cell-depleted whole blood | Tang et al., 2020^2^ |
| 7 | miR-181a-3p | Plasma | Gutmann et al., 2022^4^ |
|  |  | Red blood cell-depleted whole blood | Tang et al., 2020^2^ |
| 8 | miR-16-2-3p | Plasma | Gutmann et al., 2022^4^ |
|  |  | Red blood cell-depleted whole blood | Tang et al., 2020^2^ |
| 9 | miR-107 | Plasma | Gutmann et al., 2022^4^ |
|  |  | Red blood cell-depleted whole blood | Tang et al., 2020^2^ |
| 10 | miR-421 | Plasma | Gutmann et al., 2022^4^ |
|  |  | Red blood cell-depleted whole blood | Tang et al., 2020^2^ |
| 11 | miR-155-5p | Plasma | Gutmann et al., 2022^4^ |
|  |  | Red blood cell-depleted whole blood | Tang et al., 2020^2^ |
| 12 | miR-30e-3p | Plasma | Gutmann et al., 2022^4^ |
|  |  | Red blood cell-depleted whole blood | Tang et al., 2020^2^ |
| 13 | miR-584-5p | Plasma | Gutmann et al., 2022^4^ |
|  |  | Red blood cell-depleted whole blood | Tang et al., 2020^2^ |
| 14 | miR-598-3p | Plasma | Gutmann et al., 2022^4^ |
|  |  | Red blood cell-depleted whole blood | Tang et al., 2020^2^ |
| 15 | let-7g-5p | Plasma | Gutmann et al., 2022^4^ |
|  |  | Red blood cell-depleted whole blood | Tang et al., 2020^2^ |
| 16 | miR-342-3p | Plasma | Gutmann et al., 2022^4^ |
|  |  | Red blood cell-depleted whole blood | Tang et al., 2020^2^ |
| 17 | miR-181d-5p | Plasma | Gutmann et al., 2022^4^ |
|  |  | Red blood cell-depleted whole blood | Tang et al., 2020^2^ |
| 18 | miR-26b-5p | Plasma | Gutmann et al., 2022^4^ |
|  |  | Red blood cell-depleted whole blood | Tang et al., 2020^2^ |
| 19 | miR-98-5p | Red blood cell-depleted whole blood | Tang et al., 2020^2^ |
|  |  | Plasma | Gutmann et al., 2022^4^ |
|  |  | Plasma | Farr et al., 2021^1^ |
| 20 | miR-146b-5p | Plasma | Gutmann et al., 2022^4^ |
|  |  | Red blood cell-depleted whole blood | Tang et al., 2020^2^ |
| 21 | miR-10a-5p | Plasma | Gutmann et al., 2022^4^ |
|  |  | Red blood cell-depleted whole blood | Tang et al., 2020^2^ |
| 22 | miR-146a-5p | Plasma | Gutmann et al., 2022^4^ |
|  |  | Red blood cell-depleted whole blood | Tang et al., 2020^2^ |
| 23 | miR-145-3p | Plasma | Gutmann et al., 2022^4^ |
|  |  | Red blood cell-depleted whole blood | Tang et al., 2020^2^ |
| 24 | miR-181a-2-3p | Plasma | Gutmann et al., 2022^4^ |
|  |  | Red blood cell-depleted whole blood | Tang et al., 2020^2^ |
| 25 | miR-144-5p | Plasma | Gutmann et al., 2022^4^ |
|  |  | Red blood cell-depleted whole blood | Tang et al., 2020^2^ |
| 26 | miR-17-5p | Plasma | Gutmann et al., 2022^4^ |
|  |  | Red blood cell-depleted whole blood | Tang et al., 2020^2^ |
|  |  | Red blood cell-depleted whole blood | Tang et al., 2020^2^ |
| 27 | miR-651-5p | Plasma | Farr et al., 2021^1^ |
|  |  | Red blood cell-depleted whole blood | Tang et al., 2020^2^ |
| 28 | miR-200c-3p | Plasma | Farr et al., 2021^1^ |
|  |  | Nasopharyngeal | Tang et al., 2020^2^ |

| 29 | let-7a-3p | Plasma | Gutmann et al., 2022^4^ |
| --- | --- | --- | --- |
|  |  | Red blood cell-depleted whole blood | Tang et al., 2020^2^ |
| 30 | miR-126-5p | Plasma | Gutmann et al., 2022^4^ |
|  |  | Red blood cell-depleted whole blood | Tang et al., 2020^2^ |
| 31 | miR-142-3p | Plasma | Gutmann et al., 2022^4^ |
|  |  | Red blood cell-depleted whole blood | Tang et al., 2020^2^ |
| 32 | miR-148a-3p | Plasma | Gutmann et al., 2022^4^ |
|  |  | Red blood cell-depleted whole blood | Tang et al., 2020^2^ |
| 33 | miR-15a-5p | Plasma | Gutmann et al., 2022^4^ |
|  |  | Red blood cell-depleted whole blood | Tang et al., 2020^2^ |
| 34 | miR-16-5p | Plasma | Gutmann et al., 2022^4^ |
|  |  | Red blood cell-depleted whole blood | Tang et al., 2020^2^ |
| 35 | miR-199a-5p | Plasma | Gutmann et al., 2022^4^ |
|  |  | Red blood cell-depleted whole blood | Tang et al., 2020^2^ |
| 36 | miR-199b-5p | Plasma | Gutmann et al., 2022^4^ |
|  |  | Red blood cell-depleted whole blood | Tang et al., 2020^2^ |
| 37 | miR-20a-5p | Plasma | Gutmann et al., 2022^4^ |
|  |  | Red blood cell-depleted whole blood | Tang et al., 2020^2^ |
| 38 | miR-20b-5p | Plasma | Gutmann et al., 2022^4^ |
|  |  | Red blood cell-depleted whole blood | Tang et al., 2020^2^ |
| 39 | miR-21-3p | Plasma | Gutmann et al., 2022^4^ |
|  |  | Red blood cell-depleted whole blood | Tang et al., 2020^2^ |
| 40 | miR-21-5p | Plasma | Gutmann et al., 2022^4^ |
|  |  | Red blood cell-depleted whole blood | Tang et al., 2020^2^ |
| 41 | miR-223-3p | Plasma | Gutmann et al., 2022^4^ |
|  |  | Red blood cell-depleted whole blood | Tang et al., 2020^2^ |
| 42 | miR-223-5p | Plasma | Gutmann et al., 2022^4^ |
|  |  | Red blood cell-depleted whole blood | Tang et al., 2020^2^ |
| 43 | miR-22-3p | Plasma | Gutmann et al., 2022^4^ |
|  |  | Red blood cell-depleted whole blood | Tang et al., 2020^2^ |
| 44 | miR-22-5p | Plasma | Gutmann et al., 2022^4^ |
|  |  | Red blood cell-depleted whole blood | Tang et al., 2020^2^ |
| 45 | miR-27a-3p | Plasma | Gutmann et al., 2022^4^ |
|  |  | Red blood cell-depleted whole blood | Tang et al., 2020^2^ |
| 46 | miR-29a-3p | Plasma | Gutmann et al., 2022^4^ |
|  |  | Red blood cell-depleted whole blood | Tang et al., 2020^2^ |
| 47 | miR-320b | Red blood cell-depleted whole blood | Tang et al., 2020^2^ |
|  |  | Whole blood | Duecker et al., 2021^5^ |
| 48 | miR-32-5p | Plasma | Gutmann et al., 2022^4^ |
|  |  | Red blood cell-depleted whole blood | Tang et al., 2020^2^ |
| 49 | miR-345-5p | Plasma | Gutmann et al., 2022^4^ |
|  |  | Red blood cell-depleted whole blood | Tang et al., 2020^2^ |
| 50 | miR-423-3p | Plasma | Gutmann et al., 2022^4^ |
|  |  | Red blood cell-depleted whole blood | Tang et al., 2020^2^ |
| 51 | miR-425-5p | Plasma | Gutmann et al., 2022^4^ |
|  |  | Red blood cell-depleted whole blood | Tang et al., 2020^2^ |
| 52 | miR-486-5p | Plasma | Gutmann et al., 2022^4^ |
|  |  | Red blood cell-depleted whole blood | Tang et al., 2020^2^ |
| 53 | miR-505-3p | Plasma | Gutmann et al., 2022^4^ |
|  |  | Red blood cell-depleted whole blood | Tang et al., 2020^2^ |
| 54 | miR-7-1-3p | Plasma | Gutmann et al., 2022^4^ |
|  |  | Red blood cell-depleted whole blood | Tang et al., 2020^2^ |

Table S3.

**Dysregulated miRNAs in severe COVID-19 cases compared with non-severe ones which used for bioinformatic analysis**

| **Severe COVID-19 cases vs. Non-severe COVID-19 cases** | | | |
| --- | --- | --- | --- |
| **Number** | **miRNA** | **Tissue type** | **Reference** |
| 1 | miR-451a | Red blood cell-depleted whole blood | Tang et al., 2020^2^ |
|  |  | Plasma | Wilson et al., 2022^6^ |
|  |  | Plasma | Grehl et al., 2021^7^ |
|  |  | Plasma | Gutmann et al., 2022^4^ |
| 2 | miR-150-5p | Red blood cell-depleted whole blood | Tang et al., 2020^2^ |
|  |  | Plasma | Wilson et al., 2022^6^ |
|  |  | Plasma | Fernández-Pato et al., 2022^8^ |
| 3 | miR-584-5p | Plasma | Fernández-Pato et al., 2022^8^ |
|  |  | Red blood cell-depleted whole blood | Tang et al., 2020^2^ |
|  |  | Plasma | Gutmann et al., 2022^4^ |
| 4 | miR-1246 | Plasma | Fernández-Pato et al., 2022^8^ |
|  |  | Red blood cell-depleted whole blood | Tang et al., 2020^2^ |
|  |  | Peripheral venous blood | Parray et al^9^ |
| 5 | let-7b-5p | Red blood cell-depleted whole blood | Tang et al., 2020^2^ |
|  |  | Plasma | Wilson et al., 2022^6^ |
|  |  | Plasma | Gutmann et al., 2022^4^ |
| 6 | miR-1260a | Plasma | Fernández-Pato et al., 2022^8^ |
|  |  | Red blood cell-depleted whole blood | Tang et al., 2020^2^ |
| 7 | miR-150-3p | Plasma | Fernández-Pato et al., 2022^8^ |
|  |  | Red blood cell-depleted whole blood | Tang et al., 2020^2^ |
| 8 | miR-550b-2-5p | Plasma | Fernández-Pato et al., 2022^8^ |
|  |  | Red blood cell-depleted whole blood | Tang et al., 2020^2^ |
| 9 | miR-146a-5p | Red blood cell-depleted whole blood | Tang et al., 2020^2^ |
|  |  | Plasma | Wilson et al., 2022^6^ |
| 10 | miR-423-5p | Plasma | Fernández-Pato et al., 2022^8^ |
|  |  | Red blood cell-depleted whole blood | Tang et al., 2020^2^ |
|  |  | Plasma | Wilson et al., 2022^6^ |
| 11 | miR-185-5p | Plasma | Fernández-Pato et al., 2022^8^ |
|  |  | Plasma | Grehl et al., 2021^7^ |
|  |  | Red blood cell-depleted whole blood | Tang et al., 2020^2^ |
|  |  | Plasma | Martinez-Fleta et al., 2021^10^ |

| Table S4. | | | | | |
| --- | --- | --- | --- | --- | --- |
| **Effects of DEncRNAs on the COVID-19 according to the bioinformatics prediction and wet-lab.** | | | | | |
| **ncRNA** | **Expression direction** | **Experimental model** | **Mechanism** | **Effects** | **Reference** |
| hsa-miR-125-5p | ↓ | Bioinformatics prediction | Binds 3’UTR of ACE2 | Blocks the virus entry and attachment | ^11^ |
| hsa-miR-23b-5p | ↓ | Bioinformatics prediction | Binds 3’UTR of ACE2 | Blocks the virus entry and attachment | ^11^ |
| hsa-miR-769-5p | ↓ | Bioinformatics prediction | Binds 3’UTR of ACE2 | Blocks the virus entry and attachment | ^11^ |
| hsa-miR-98 | ↓ | Bioinformatics prediction | Binds 3’UTR of S protein | Blocks the virus entry and attachment | ^12^ |
| hsa-miR-622 | ↓ | Bioinformatics prediction | Binds 3’UTR of S protein | Blocks the virus entry and attachment | ^13^ |
| hsa-miR-761 | ↓ | Bioinformatics prediction | Binds 3’UTR of S protein | Blocks the virus entry and attachment | ^13^ |
| hsa-miR-15b-5p | ↓ | Bioinformatics prediction | Binds 3’UTR of S protein | Blocks the virus entry and attachment | ^13^ |
| hsa-miR-338-3p | ↓ | Bioinformatics prediction | Binds 3’UTR of S protein | Blocks the virus entry and attachment | ^13^ |
| hsa-miR-4462 | ↓ | Bioinformatics prediction | Binds 3’UTR of S protein | Blocks the virus entry and attachment | ^13^ |
| hsa-miR-4464 | ↓ | Bioinformatics prediction | Binds 3’UTR of S protein | Blocks the virus entry and attachment | ^13^ |
| hsa-miR-7107-5p | ↓ | Bioinformatics prediction | Binds 3’UTR of S protein | Blocks the virus entry and attachment | ^13^ |
| hsa-miR-885-5p | ↓ | Bioinformatics prediction | Binds 3’UTR of S protein | Blocks the virus entry and attachment | ^13^ |
| hsa-miR-5187-5p | ↓ | Bioinformatics prediction | Binds 3’UTR of S protein | Blocks the virus entry and attachment | ^13^ |
| hsa-miR-4661-3p | ↓ | Bioinformatics prediction | Binds 3’UTR of S protein | Blocks the virus entry and attachment | ^14^ |
| hsa-miR-148a-3p | ↑ | Bioinformatics prediction | Targets the ORF1a, E, S and M genes | Regulates virus infection | ^15^ |
| hsa-miR-497-5p | ↓ | Bioinformatics prediction | Targets the ssRNA of viral coding strand | Inhibits viral replication | ^16,17^ |
| hsa-miR-21-3p | ↓ | Bioinformatics prediction | Targets the ssRNA of viral coding strand | Inhibits viral replication | ^16,17^ |
| hsa-miR-195-5p | ↓ | Bioinformatics prediction | Targets the ssRNA of viral coding strand | Inhibits viral replication | ^16,17^ |
| hsa-miR-17-3p | ↑ | Bioinformatics prediction | Its mRNA target (*DICER*) is significantly decreased. | Regulates inflammatory miRNAs and immune system | ^18^ |
| hsa-miR-776-3p | ↓ | Bioinformatics prediction | Inhibits the expression of IL-6 | Anti-inflammatory | ^1^ |
| hsa-miR-486-5p | ↓ | Bioinformatics prediction | Targets the gene OTUD7B | Regulates antiviral response and promotes acute lung injury | ^15^ |
| hsa-miR-29a-3p | ↓ | Bioinformatics prediction | Regulates COL5A3 expression | Regulates inflammatory response | ^18^ |
| hsa-miR-31-3p | ↓ | Bioinformatics prediction | Regulates ZMYM5 expression | Regulates inflammatory response | ^18^ |
| hsa-miR-126-3p | ↓ | Bioinformatics prediction | Regulates CAMSAP1 expression | Regulates inflammatory response and increases ARDS vulnerability | ^18^ |
| hsa-miR-146a-5p | ↓ | Bioinformatics prediction | Targets TRAF6 and IRAK1 and regulates NF-κB and IL-6 expression | Regulates inflammatory response | ^19^ |
| hsa-miR-21-5p | ↓ | Bioinformatics prediction | Involves in NF-κB signaling pathway | Inhibits inflammatory response | ^19^ |
| hsa-miR-21 | ↓ | Bioinformatics prediction | Regulation of IL-12 and p53 expression | Anti-neuroinflammatory activity | ^20^ |
| hsa-miR-124 | ↓ | Bioinformatics prediction | Regulation of Stat3 expression | Anti-neuroinflammatory activity | ^20^ |
| hsa-miR-146a | ↓ | Bioinformatics prediction | Regulation of TRAF6 expression | Anti-neuroinflammatory activity | ^20^ |
| hsa-miR-326 | ↑ | Bioinformatics prediction | Regulation of CEBPA expression | Pro-neuroinflammatory activity | ^20^ |
| hsa-miR-155 | ↑ | Bioinformatics prediction | Regulation of SOCS1 expression | Pro-neuroinflammatory activity | ^20^ |
| hsa-miR-27b | ↑ | Bioinformatics prediction | Regulation of PPARS expression | Pro-neuroinflammatory activity | ^20^ |
| hsa-miR-451a | ↓ | Bioinformatics prediction | Promotes expression of IL-6R | Promotes cytokine storm | ^21^ |
| hsa-miR-374a | ↓ | Bioinformatics prediction | Targets the CCL2 | Promotes cytokine storm and ARDS | ^21^ |
| hsa-miR-10b | ↓ | Bioinformatics prediction | Increases levels of IL-2 and LI-8 | Promotes cytokine storm | ^22^ |
| hsa-miR-155 | ↓ | Bioinformatics prediction | Targets SHIP1 and SOCS | Regulates inflammatory response and antiviral response | ^23^ |
| hsa-miR-146b | ↑ | Bioinformatics prediction | Targets IL-6, IL-8, IRAK1 and TRAF6 | Regulates inflammatory response | ^23^ |
| hsa-miR-146a | ↑ | Bioinformatics prediction | Targets IL-6, IL-8, IRAK1 and TRAF6 | Regulates inflammatory response | ^23^ |
| hsa-miR-499 | ↑ | Bioinformatics prediction | Targets SOX6 | Regulates inflammatory response | ^23^ |
| hsa-miR-146a-5p | ↓ | Bioinformatics prediction | Targets STAT1 | Regulates host immune response | ^23^ |
| hsa-miR-21-5p | ↓ | Bioinformatics prediction | Targets CCL20 and IRAK1 | Regulates host immune response | ^24^ |
| hsa-miR-486-3p | ↑ | Bioinformatics prediction | Targets MAF | Induces immune response | ^24^ |
| hsa-miR-486-5p | ↑ | Bioinformatics prediction | Targets NRP2 | Regulates inflammatory response | ^24^ |
| hsa-miR-181a-2-3p | ↓ | Bioinformatics prediction | Associated with CXCL8 and TLR4 expression | Involves in inflammatory response and chronic obstructive pulmonary disease | ^24^ |
| hsa-miR-99a-5p | ↓ | Bioinformatics prediction | Targets IGF1R and MTMR3 | Promotes antiviral immunity | ^24^ |
| hsa-miR-146a-5p | ↓ | Bioinformatics prediction | Targets TRAF6 | Regulates inflammatory response and thrombosis | ^25^ |
| hsa-miR-133a | ↑ | Bioinformatics prediction | Regulates neutrophil counts and degranulation | Involves in inflammation induced myocyte damage | ^4^ |
| hsa-miR-483-5p | ↑ | Bioinformatics prediction | Targets IGF1 | Acts as targets in cardiometabolic disease | ^26^ |
| GATA5 | ↑ | Bioinformatics prediction | Inhibits ACE2 gene expression | Prevents virus entry into cells | ^27^ |
| WAKMAR2 | ↑ | Bioinformatics prediction | Regulates cytokine signaling pathway | Regulates viral replication | ^28^ |
| EGOT | ↑ | Bioinformatics prediction | Regulates cytokine signaling pathway | Regulates viral replication | ^28^ |
| NEAT1 | ↓ | Bioinformatics prediction | Induces TNF-α, IL-6 and IL-1 | Regulates neutrophils chemotaxis and induced inflammatory injury | ^29^ |
|  | ↑ | Bronchial epithelial cells | Downregulates CAPN1 | Regulates inflammatory development | ^29,30^ |
| MALAT1 | ↓ | Bioinformatics prediction | Induces TNF-α, IL-6 and IL-1 | Regulates neutrophils chemotaxis and induced inflammatory injury | ^29^ |
|  | ↑ | BALF | Downregulates IL-8 and CAPN1 | Regulates inflammatory injury and promote acute lung injury | ^30-33^ |
| AC009088 | ↑ | Bioinformatics prediction | Downregulates Pycard protein | Inhibits Pycard transcription | ^31^ |
| LINC02384 | ↓ | Bioinformatics prediction | Regulates IFN-γ | Regulates antiviral response and innate immune response | ^31^ |
| HOTAIRM1 | ↓ | Bioinformatics prediction | Regulates IL-17 signaling pathway | Regulates inflammatory development | ^31^ |
| PVT1 | ↑ | Bioinformatics prediction | Regulates IL-17 signaling pathway | Regulates inflammatory development | ^31^ |
| SNHG25 | ↓ | Bioinformatics prediction | Induces TNF-α, IL-6 and IL-1 | Regulates neutrophils chemotaxis and induced inflammatory injury | ^29^ |
| HIF1A-AS-1 | ↑ | Bioinformatics prediction | Regulates the interaction between HIF1α and Jun | Regulates T cell differentiation and clearance of virus | ^34^ |
| RORA-AS-7 | ↑ | Bioinformatics prediction | Regulates the interaction between RORA and AP-1 | Regulates T cell differentiation | ^34^ |
| GAS5 | ↑ | Bioinformatics prediction | Upregulates IL-10 and TNF-α | Regulates antiviral response and decrease lipopolysaccharide | ^35^ |
| NRAV | ↓ | Bioinformatics prediction | Increases CCL2 and CCL3 | Regulates host immune response and inhibits viral replication | ^35^ |
| TUG1 | ↑ | Bioinformatics prediction | Increases IL-7 and CCL2 | Regulates inflammatory development | ^35^ |
| NORAD | ↑ | Bioinformatics prediction | Increases IL-6, TNF-α, CSF3 and CXCL10 | Promotes cytokine storm | ^35^ |
| RAD51-AS1 | ↑ | Bioinformatics prediction | Increases IL-6, TNF-α and CCL2 | Promotes pro-inflammatory immune response and cytokine storm | ^35^ |
| DANCR | ↓ | Lung tissues | Increases REL, RELA and NF-κB1 | Promotes infection response | ^36^ |
| SMC2-AS1 | ↑ | Bioinformatics prediction | Regulates Wnt and TGF-β signaling pathway | Regulates lung repair and regeneration | ^24,30,37,38^ |
| AL392172 | ↑ | Bioinformatics prediction | Regulates IL-17 signaling pathway | Regulates viral transcription and inflammation | ^31^ |
| TTTY15 | ↑ | Bioinformatics prediction | Promotes T-box transcription factor 4 | Regulates cell infection | ^39,40^ |
| SNHG1 | ↑ | Bioinformatics prediction | Increases IL-10 and CCL2 | Regulates immune response and cytokine storm | ^41^ |
| MEG3 | ↑ | Bioinformatics prediction | Acts as sponge of miR-223 and enhances the expression of NLRP3 | Activates NF-κB signaling pathway and cytokine storm, and regulates virus replication progression | ^42^ |
| KCNQ1OT1 | ↑ | Bioinformatics prediction | Acts as sponge of miR-let-7c and miR-let-7f and increases expression of IL-6, TNFR-II and NLRP3 | Increases virus entry and inflammation | ^43^ |
| Ppp1r10 | ↓ | Bioinformatics prediction | Acts as sponges for miR-124-3p, impedes Ddx58 degradation | Inhibits virus replication | ^44^ |
| C330019G07Rik | ↓ | Bioinformatics prediction | Acts as sponges for miR-124-3p, impedes Ddx58 degradation | Inhibits virus replication | ^44^ |
| hsa_circ_0000566 | ↓ | Bioinformatics prediction | Binds with RBPs including AGO2, ELF4A3, HuR, U2AF65 whose parent gene is VRK1 | Regulates viral infection, inflammatory response and immune response | ^45^ |
| hsa_circ_0001681 | ↓ | Bioinformatics prediction | Binds with RBPs including EIF4A3 and FUS whose parent gene is RAPGEF5 | Regulates viral infection, inflammatory response and immune response | ^45^ |
| hsa_circ_0080941 | ↑ | Bioinformatics prediction | Binds with EIF4A3 whose parent gene is PCLO | Regulates viral infection, inflammatory response and immune response | ^45^ |
| hsa_circ_0080942 | ↑ | Bioinformatics prediction | Binds with EIF4A3 and FUS whose parent gene is PCLO | Regulates viral infection, inflammatory response and immune response | ^45^ |
| hsa_circ_0005630 | ↓ | Bioinformatics prediction | Binds with EIF4A3 whose parent gene is RAB11FIP1 | Regulates viral infection, inflammatory response and immune response | ^45^ |
| hsa_circ_3205 | ↑ | Bioinformatics prediction | Interacts with miR-298 and contribute to the upregulation of KCNMB4 and PRKCE | Regulates blood coagulation and immune response and cytokine storm | ^46^ |
| circFNDC3B | ↑ | Bioinformatics prediction | Interacts with AGO protein, leading to the dysregulated biogenesis of miRNAs | Activates pro-inflammatory response and cytokine storm | ^47^ |
| circCNOT1 | ↑ | Bioinformatics prediction | Interacts with AGO protein, leading to the dysregulated biogenesis of miRNAs | Activates pro-inflammatory response and cytokine storm | ^47^ |
| hsa_circ_100782 | ↑ | Bioinformatics prediction | Acts as sponge of hsa-miR-124 and increases IL-6R and STAT3 | Activates NF-κB signaling pathway and cytokine storm development | ^48^ |
| hsa_circ_0080942 | - | Bioinformatics prediction | As ceRNA of hsa-miR-486-3p targeting IL33, IL13, IL1B and IL7 | Regulates cytokine storm | ^49^ |
| hsa_circ_0080135 | - | Bioinformatics prediction | As ceRNA of hsa-miR-769-3p targeting IL12B, IFNG, CXCL6 and CXCL8 | Regulates cytokine storm | ^49^ |
| hsa_circ_0000479 |  | Bioinformatics prediction | As ceRNA of hsa-miR-149-5p targeting RIG-I and IL-6 | Regulates immune response | ^50^ |

**References**

1 Farr, R. J. *et al.* Altered microRNA expression in COVID-19 patients enables identification of SARS-CoV-2 infection. *PLoS Pathog.* **17**, e1009759 (2021).

2 Tang, H. *et al.* The noncoding and coding transcriptional landscape of the peripheral immune response in patients with COVID-19. *Clin. Transl. Med.* 10, e200 (2020).

3 Akula, S. M., Bolin, P. & Cook, P. P. Cellular miR-150-5p may have a crucial role to play in the biology of SARS-CoV-2 infection by regulating nsp10 gene. *RNA Biol.* **19**, 1-11 (2022).

4 Gutmann, C. *et al.* Association of cardiometabolic microRNAs with COVID-19 severity and mortality. *Cardiovasc. Res.* **118**, 461-474 (2022).

5 Duecker, R. P. *et al.* The MiR-320 family is strongly downregulated in patients with COVID-19 induced severe respiratory failure. *Int. J. Mol. Sci.* **22,** 10351 (2021).

6 Wilson, J. C. *et al.* Integrated miRNA/cytokine/chemokine profiling reveals severity-associated step changes and principal correlates of fatality in COVID-19. *iScience* **25,** 103672 (2022).

7 Grehl, C. *et al.* Detection of SARS-CoV-2 derived small rnas and changes in circulating small rnas associated with COVID-19. *Viruses* **13,** 1593 (2021).

8 Fernandez-Pato, A. *et al.* Plasma miRNA profile at COVID-19 onset predicts severity status and mortality. *Emerg Microbes Infect.* **11**, 676-688 (2022).

9 Parray, A. *et al.* SnoRNAs and miRNAs networks underlying COVID-19 disease severity. *Vaccines* **9,** 1056 (2021).

10 Martínez-Fleta, P. *et al.* A differential signature of circulating miRNAs and cytokines between COVID-19 and community-acquired pneumonia uncovers novel physiopathological mechanisms of COVID-19. *Front. Immunol.* **12**, 815651 (2021).

11 Nersisyan, S. *et al.* Potential role of cellular miRNAs in coronavirus-host interplay. *PeerJ.* **8**, e9994 (2020).

12 Matarese, A., Gambardella, J., Sardu, C. & Santulli, G. miR-98 Regulates TMPRSS2 expression in human endothelial cells: key implications for COVID-19. *Biomedicines* **8,** 462 (2020).

13 Sardar, R., Satish, D., Birla, S. & Gupta, D. Integrative analyses of SARS-CoV-2 genomes from different geographical locations reveal unique features potentially consequential to host-virus interaction, pathogenesis and clues for novel therapies. *Heliyon* **6**, e04658 (2020).

14 Arghiani, N., Nissan, T. & Matin, M. M. Role of microRNAs in COVID-19 with implications for therapeutics. *Biomed. Pharmacother.* **144**, 112247 (2021).

15 de Gonzalo-Calvo, D. *et al.* Circulating microRNA profiles predict the severity of COVID-19 in hospitalized patients. *Transl. Res.* **236**, 147-159 (2021).

16 Chen, L. & Zhong, L. Genomics functional analysis and drug screening of SARS-CoV-2. *Genes. Dis.* **7**, 542-550 (2020).

17 Cong, Y. *et al.* Nucleocapsid protein recruitment to replication-transcription complexes plays a crucial role in coronaviral life cycle. *J. Virol.* **94,** e01925-19 (2020).

18 Keikha, R., Hashemi-Shahri, S. M. & Jebali, A. The relative expression of miR-31, miR-29, miR-126, and miR-17 and their mRNA targets in the serum of COVID-19 patients with different grades during hospitalization. *Eur. J. Med. Res.* **26**, 75 (2021).

19 Sabbatinelli, J. *et al.* Decreased serum levels of the inflammaging marker miR-146a are associated with clinical non-response to tocilizumab in COVID-19 patients. *Mech. Ageing. Dev.* **193**, 111413 (2021).

20 Keikha, R., Hashemi-Shahri, S. M. & Jebali, A. The miRNA neuroinflammatory biomarkers in COVID-19 patients with different severity of illness. *Neurologia. (Engl Ed)* **38**, e41-e51 (2023).

21 Yang, P. *et al.* Downregulated miR-451a as a feature of the plasma cfRNA landscape reveals regulatory networks of IL-6/IL-6R-associated cytokine storms in COVID-19 patients. *Cell. Mol. Immunol.* **18**, 1064-1066 (2021).

22 Bagheri-Hosseinabadi, Z. *et al.* The relationship between serum levels of interleukin-2 and IL-8 with circulating microRNA-10b in patients with COVID-19. *Iran. J. Immunol.* **18**, 65-73 (2021).

23 Kassif-Lerner, R. *et al.* miR-155: A potential biomarker for predicting mortality in COVID-19 Patients. *J. Pers. Med.* **12,** 324 (2022).

24 Tang, H. *et al.* The noncoding and coding transcriptional landscape of the peripheral immune response in patients with COVID-19. *Clin. Transl. Med.* **10**, e200 (2020).

25 Martinez-Fleta, P. *et al.* A differential signature of circulating mirnas and cytokines between COVID-19 and community-acquired pneumonia uncovers novel physiopathological mechanisms of COVID-19. *Front. Immunol.* **12**, 815651 (2021).

26 Giuliani, A. *et al.* Circulating miR-320b and miR-483-5p levels are associated with COVID-19 in-hospital mortality. *Mech. Ageing Dev.* **202**, 111636 (2022).

27 Cheng, J. *et al.* Risk stratification by long non-coding RNAs profiling in COVID-19 patients. *J. Cell Mol. Med.* **25**, 4753-4764 (2021).

28 Mukherjee, S., Banerjee, B., Karasik, D. & Frenkel-Morgenstern, M. mRNA-lncRNA Co-expression network analysis reveals the role of lncRNAs in immune dysfunction during severe SARS-CoV-2 infection. *Viruses* **13,** 402 (2021).

29 Shaath, H., Vishnubalaji, R., Elkord, E. & Alajez, N. M. Single-cell transcriptome analysis highlights a role for neutrophils and inflammatory macrophages in the pathogenesis of severe COVID-19. *Cells* **9,** 2374 (2020).

30 Vishnubalaji, R., Shaath, H. & Alajez, N. M. Protein coding and long noncoding RNA (lncRNA) transcriptional landscape in SARS-CoV-2 infected bronchial epithelial cells highlight a role for interferon and inflammatory response. *Genes (Basel)* **11,** 760 (2020).

31 Moazzam-Jazi, M. *et al.* Interplay between SARS-CoV-2 and human long non-coding RNAs. *J. Cell. Mol. Med.* **25**, 5823-5827 (2021).

32 Wei, L. *et al.* Silencing of lncRNA MALAT1 prevents inflammatory injury after lung transplant ischemia-reperfusion by downregulation of IL-8 via p300. *Mol. Ther. Nucleic Acids.* **18**, 285-297 (2019).

33 Li, H. *et al.* BML-111 alleviates acute lung injury through regulating the expression of lncRNA MALAT1. *Arch. Biochem. Biophys.* **649**, 15-21 (2018).

34 Zheng, H. Y. *et al.* Longitudinal transcriptome analyses show robust T cell immunity during recovery from COVID-19. *Signal Transduct. Target. Ther.* **5**, 294 (2020).

35 Morenikeji, O. B. *et al.* Evolutionarily conserved long non-coding RNA regulates gene expression in cytokine storm during COVID-19. *Front. Bioeng. Biotechnol.* **8**, 582953 (2020).

36 Meydan, C., Madrer, N. & Soreq, H. The neat dance of COVID-19: NEAT1, DANCR, and co-modulated cholinergic RNAs link to inflammation. *Front. Immunol.* **11**, 590870 (2020).

37 Yousefi, H. *et al.* SARS-CoV infection crosstalk with human host cell noncoding-RNA machinery: An in-silico approach. *Biomed. Pharmacother.* **130**, 110548 (2020).

38 Villar, J., Zhang, H. & Slutsky, A. S. Lung repair and regeneration in ARDS: Role of PECAM1 and Wnt signaling. *Chest.* **155**, 587-594 (2019).

39 Lai, I. L. *et al.* Male-specific long noncoding RNA TTTY15 inhibits non-small cell lung cancer proliferation and metastasis via TBX4. *Int. J. Mol. Sci.* **20,** 3473 (2019).

40 Aishwarya, S., Gunasekaran, K. & Margret, A. A. Computational gene expression profiling in the exploration of biomarkers, non-coding functional RNAs and drug perturbagens for COVID-19. *J. Biomol. Struct. Dyn.* **40**, 3681-3696 (2022).

41 Kesheh, M. M., Mahmoudvand, S. & Shokri, S. Long noncoding RNAs in respiratory viruses: A review. *Rev. Med. Virol.* **32**, e2275 (2022).

42 Zhang, Y. *et al.* Melatonin prevents endothelial cell pyroptosis via regulation of long noncoding RNA MEG3/miR-223/NLRP3 axis. *J. Pineal. Res.* **64,** 12449 (2018).

43 Turjya, R. R., Khan, M. A. & Mir Md Khademul Islam, A. B. Perversely expressed long noncoding RNAs can alter host response and viral proliferation in SARS-CoV-2 infection. *Future Virol.* **15**, 577-593 (2020).

44 Arora, S. *et al.* Unravelling host-pathogen interactions: CeRNA network in SARS-CoV-2 infection (COVID-19). *Gene.* **762**, 145057 (2020).

45 Yang, M. *et al.* Differential host circRNA expression profiles in human lung epithelial cells infected with SARS-CoV-2. *Infect. Genet. Evol.* **93**, 104923 (2021).

46 Barbagallo, D. *et al.* Competing endogenous RNA network mediated by circ_3205 in SARS-CoV-2 infected cells. *Cell. Mol. Life. Sci.* **79**, 75 (2022).

47 Zhang, X. *et al.* Competing endogenous RNA network profiling reveals novel host dependency factors required for MERS-CoV propagation. *Emerg Microbes. Infect.* **9**, 733-746 (2020).

48 Chen, G., Shi, Y., Zhang, Y. & Sun, J. CircRNA_100782 regulates pancreatic carcinoma proliferation through the IL6-STAT3 pathway. *Onco. Targets Ther.* **10**, 5783-5794 (2017).

49 Ayaz, H. *et al.* Mapping CircRNA-miRNA-mRNA regulatory axis identifies hsa_circ_0080942 and hsa_circ_0080135 as a potential theranostic agents for SARS-CoV-2 infection. *PLoS. One.* **18**, e0283589 (2023).

50 Firoozi, Z. *et al.* Hsa_circ_0000479/Hsa-miR-149-5p/RIG-I, IL-6 Axis: A potential novel pathway to regulate immune response against COVID-19. *Can. J. Infect. Dis. Med. Microbiol.* **2022**, 2762582 (2022).
